# CaMYBA–CaMYC–CaTTG1 complex activates the transcription of anthocyanin synthesis structural genes and regulates anthocyanin accumulation in pepper (*Capsicum annuum* L.) leaves

**DOI:** 10.3389/fpls.2025.1538607

**Published:** 2025-03-07

**Authors:** Xiaowei Ma, Guangbo Liang, Ziqian Xu, Chenwei Lin, Biao Zhu

**Affiliations:** Key Laboratory of Quality and Safety Control for Subtropical Fruit and Vegetable, Ministry of Agriculture and Rural Affairs, Collaborative Innovation Center for Efficient and Green Production of Agriculture in Mountainous Areas of Zhejiang Province, College of Horticulture Science, Zhejiang A&F University, Hangzhou, Zhejiang, China

**Keywords:** pepper, anthocyanin, MBW complex, regulatory complex, transcriptional regulation

## Abstract

Anthocyanins are flavonoid-derived metabolites that contribute to plant and human health. At present, few studies have studied the biosynthesis and accumulation mechanism of anthocyanins in pepper leaves. The role of CaMYBA–CaMYC–CaTTG1 complex in anthocyanin biosynthesis in pepper leaves was studied. Yeast two-hybrid and dual-luciferase experiments showed that CaMYBA, CaMYC, and CaTTG1 could form an MYB–bHLH–WD40 (MBW) complex. They also have transcriptional activation on the anthocyanin synthesis structural genes *CaCHS*, *CaCHI*, *CaF3H*, *CaF3′5′H*, *CaANS*, *CaDFR*, and *CaUFGT*. Silencing *CaMYBA* or *CaMYC* could decrease the content of anthocyanin in pepper leaves. Transient overexpression of *CaMYBA* in tobacco indicated that CaMYBA determines the function of an MBW complex. Further analysis showed that CaMYBA could activate the expression of *CaMYC* by binding to its promoter. Overall, our study expands the understanding of the regulatory mechanism of anthocyanin synthesis in pepper leaves and has important significance for creating more pepper plants with different color patterns by gene editing engineering.

## 1 Introduction

Anthocyanins are one of the important factors that produce a series of colors in different tissue parts of plants such as leaves, flowers, and fruits. Anthocyanins are a major branch of flavonoid metabolism whose biological functions are diverse and play important roles in plant metabolism and breeding, including attracting insects and birds to pollinate and spread seeds and preventing photooxidative damage (Feild et al., 2001; Liu et al., 2020; Winkel-Shirley, 2001). In recent years, researchers have paid more and more attention to anthocyanin synthesis and its regulatory mechanism, which has been well studied in petunia (Albert et al., 2014), snapdragons (Naing et al., 2017), maize (Chachar et al., 2024), and *Arabidopsis* (Gonzalez et al., 2008).

The anthocyanin biosynthetic pathway branches off from the general phenylpropanoid pathway (Borovsky et al., 2004), and its synthesis requires two categories of genes: those encoding the enzymes that catalyze different reactions (i.e., structural genes) and those that regulate the expression of the structural genes (i.e., regulatory genes) (Gonzali et al., 2009). All structural genes in the pathway, including chalcone synthase (CHS), chalcone isomerase (CHl), flavanone 3-hydroxylase (F3H), flavonoid 3′5′-hydroxylase (F3′5′H), dihydroflavonol-4-reductase (DFR), anthocyanin synthase (ANS), and UDP-glucose:flavonoid 3-glucosyltransferase (UFGT), have been identified in some crops (Lalusin et al., 2006; Sunil and Shetty, 2022). *CHS*, *CHI*, and *F3H* are early biosynthetic genes (EBGs), while *F3′5′H*, *DFR*, *ANS*, and *UFGT* are late biosynthetic genes (LBGs) (Jung et al., 2019).

These structural genes usually are regulated by multiple transcription factors, especially MYB, bHLH, and WD40 proteins (Petroni and Tonelli, 2011). The three transcription factors can form a regulatory complex MYB–bHLH–WD40 (MBW) and interact with the promoters of the structural genes of the anthocyanin biosynthesis pathway to regulate their expression (Petroni and Tonelli, 2011; Spelt et al., 2000). For example, the AtMYB113/AtMYB114/AtPAP1/AtPAP2-AtEGL3/AtGL3-AtTTG1 complex can control the accumulation of anthocyanin in *Arabidopsis*, overexpression of AtMYB113 or AtMYB114 results in substantial increases in pigment production similar to the result of overexpression of AtPAP1 or AtPAP2, and pigment production in these overexpressors remains TTG1- and bHLH-dependent (Gonzalez et al., 2008). In *Petunia*, the MYB–bHLH–WD40 complex regulates anthocyanin biosynthesis at different tissue sites, and different MYBs determine the tissue-specific accumulation of anthocyanins (Albert et al., 2011). The patterning and spatial localization of anthocyanins are primarily determined by the activity of the R2R3-MYB factors in the complex, with individual gene-family members regulating separate patterns (Davies et al., 2012), which act with common bHLH and WD40 factors (Schwinn et al., 2006; Gonzalez et al., 2008; Albert et al., 2011; Lowry et al., 2012).

Pepper (*Capsicum annuum* L.) is one of the most important horticultural crops due to its culinary and ornamental applications (Liu et al., 2020). Anthocyanin-rich pepper cultivars can become special peppers with high antioxidant activity (Sharma et al., 2016). Anthocyanin pigmentation in *C. annuum* is influenced by the locus *A*, which encodes a MYB transcription factor (CaMYBA). The expression of EBGs (*CaCHS* and *CaCHI*) and LBGs (*CaDFR* and *CaANS*) of the anthocyanin pathway in pepper has been proposed to be *A*-independent (Borovsky et al., 2004). The insertion of a non-long terminal repeat (non-LTR) retrotransposon in the *CaMYBA* promoter region causes it to recruit transcription factors to activate *CaMYBA* expression, resulting in purple pigmentation in various tissues including fruits (only at the immature stage), flowers, and leaves (Jung et al., 2019). However, tissue-specific anthocyanin pigmentation is still present in some capsicum materials that carry the non-functional *CaMYBA* allele, especially in flowers and fruits. *CaAN3* can induce capsicum fruit-specific anthocyaninosis (Byun et al., 2022). The mutation of WD40 transcription factor CaTTG1 can lead to the disappearance of the hypocotyl anthocyanin phenotype of pepper (Wang et al., 2025). A recessive gene *ayw*, which encodes F3′5′H, was identified as the major candidate gene influencing the yellow color of the anthers and the green color of the stems after preliminary and fine mapping (Wang et al., 2023). Previously, virus-induced gene silencing (VIGS) of the *CaMYBA* gene induced the downregulation of most anthocyanins that synthesize structural genes (Aguilar-Barragán and Ochoa-Alejo, 2014; Zhang et al., 2015) and bHLH gene *CaMYC* (Zhang et al., 2015). However, VIGS of *CaMYC* also reduced the expression levels of most anthocyanins that synthesize structural genes, but not *CaMYBA* (Lu et al., 2019). VIGS of *CaTTG1* reduced the expression levels of some anthocyanins that synthesize structural genes (Aguilar-Barragán and Ochoa-Alejo, 2014). Although some studies have reported that *CaMYBA*, *CaMYC*, and *CaTTG1* are highly correlated with anthocyanin synthesis in pepper, they may form an MBW complex to regulate the transcriptional expression of anthocyanin synthesis structural genes. However, whether they can form an MBW complex or their specific molecular mechanism of regulating anthocyanin synthesis has not been deeply studied. This study verified that *CaMYBA*, *CaMYC*, and *CaTTG1* can form an MBW complex and then regulate the transcriptional expression of *CaCHS*, *CaCHI*, *CaF3H*, *CaF3′5′H*, *CaDFR*, *CaANS*, and *CaUFGT*, thus affecting anthocyanin synthesis in pepper leaves.

## 2 Materials and methods

### 2.1 Plant material

Green pepper (Zunla) and purple pepper (PP) were used as experimental materials. The whole plant of PP material was purple. Its stems, leaves, and flowers were purple, and its young fruit was purple, which gradually changed to orange after the fruit ripened. The materials treated in the same phase were randomly selected and divided into three groups as three biological replicates. Mature leaves of different colors were taken, immediately frozen in liquid nitrogen, and stored at −80°C for further analysis.

### 2.2 Virus-induced gene silencing of *CaMYBA* and *CaMYC*

According to the method of Wang et al. (2013a), gene-specific primers with restriction enzyme cleavage sites were used to amplify *CaMYBA* and *CaMYC* coding region fragments (**Supplementary Table S1**). The obtained product was inserted into the pTRV2 carrier to generate the pTRV2:CaMYBA and pTRV2:CaMYC. pTRV1, pTRV2, pTRV2:CaMYBA, and pTRV2:CaMYC vectors were transformed into *Agrobacterium tumefaciens* strain (GV3101). GV3101 carrying pTRV1 was mixed with the empty vector pTRV2:00, pTRV2:CaMYBA, and pTRV2:CaMYC in a 1:1 ratio. When the first set of true leaves fully unfolded, the *Agrobacterium* suspension containing pTRV1, pTRV2:00, pTRV2:CaMYBA, and pTRV2:CaMYC (OD600 = 1.0) was soaked into the fully developed cotyledons of PP using a 1.0-mL sterile needle-free syringe. The *Agrobacterium*-inoculated pepper plants were grown for 54 hours under conditions of 16°C, 75% relative humidity, and darkness and then transferred to a growth chamber at 22°C, 60% relative humidity, and 16 hours of light/8 hours of dark light cycle.

### 2.3 Yeast two-hybrid assay

Yeast two-hybrid (Y2H) assay was conducted according to the study of Wei et al. (2020). For yeast two-hybrid assay, the Coding sequences (CDSs) of CaMYBA (NM_001324618.1), CaMYC (XM_016686645.2), and CaTTG1 (XM_016708729.2) were cloned into the pGADT7 and pGBKT7 vectors to generate the prey and bait plasmids (**Supplementary Table S1**). The recombinant plasmids were co-transformed into the Y2H GOLD strain and grown on SD/-Leu/-Trp medium at 30°C for 3–4 days. Then, several dilutions of transformants were transferred to SD/-Leu/-Trp/-His/-Ade with X-α-Gal.

### 2.4 Luciferase complementation assay

According to the method of Luo et al. (2020), the full lengths of *CaMYBA*, *CaMYC*, and *CaTTG1* were inserted into the pCAMBIA1300-nLUC and pCAMBIA1300-cLUC vectors (**Supplementary Table S1**). The constructs and empty plasmids were transformed into GV3101 and transiently expressed in *Nicotiana benthamiana* leaves. The LUC fluorescence signal was observed through a Tanon 5200 imaging system (Tanon, Shanghai, China).

### 2.5 Bimolecular fluorescence complementation assay

Full-length coding sequences of CaMYBA, CaMYC, and CaTTG1 were cloned into the binary N-terminal fragment of yellow fluorescent protein (nYFP) and C-terminal fragment of yellow fluorescent protein (cYFP) vectors (**Supplementary Table S1**). According to the method of Wang et al. (2021), *Agrobacterium* strains transformed with indicated nYFP or cYFP vectors were incubated, harvested, and resuspended in infiltration buffer (0.2 mM acetosyringone, 10 mM MgCl_2_, and 10 mM MES, pH 5.6) to identical concentrations (OD600 = 0.8). Equal volumes of an *Agrobacterium* culture containing nYFP (OD600 = 0.8) and cYFP (OD600 = 0.8) were mixed before infiltration into *N. benthamiana* leaves. After infiltration, plants were incubated at 24°C for 48 hours before observation. A confocal laser scanning microscope was used to identify yellow fluorescent protein (YFP) and 4',6-diamidino-2-phenylindole (DAPI) fluorescent signals.

### 2.6 Dual-luciferase assay

The CDSs of CaMYBA, CaMYC, and CaTTG1 were amplified and ligated into the pGreenII-62-SK vector for the generation of effector constructs (**Supplementary Table S1**). The *CaCHS*, *CaCHI*, *CaF3H*, *CaF3′5′H*, *CaDFR*, *CaANS*, and *CaUFGT* promoter fragments were ligated into the pGreenII-0800-LUC vector as reporters (**Supplementary Tables S1** and **S2**). According to the method of Luo et al. (2020), the effector and reporter constructs were introduced into *A. tumefaciens* strain GV3101 (pSoup-p19) and transiently expressed in 4-week-old tobacco leaves as previously described. The luminescent living image was obtained using the plant living imaging system after 48 hours of incubation at 24°C following infiltration. The real-time fluorescence technique was employed for the quantitative assessment of LUC and REN expression, as well as for the calculation of their ratio to determine transcriptional activity, and the control ratio was set to 1.

### 2.7 Determination of anthocyanins

The content of anthocyanins was determined following the methods described in a previous study (Zhang et al., 2020). The samples were ground into powder with liquid nitrogen, followed by 24 hours of extraction in 1% HCl–methyl alcohol (3 mL, v/v) under 4°C in the dark. The absorbance at 530 nm and 657 nm was determined, and estimation of anthocyanin concentration was conducted based on the following formula: Q Anthocyanins = (A_530_ − 0.25 * A_657_)/fresh weight.

### 2.8 RNA extraction and real-time quantitative RT-PCR

Total RNA (1 μg), extracted using TRIzol reagent (Sangon Biotech, Shanghai, China), was used for oligo (dT) 18-primed cDNA synthesis according to the reverse transcription protocol (Vazyme, Nanjing, China). The resulting cDNA was subjected to real-time quantitative RT-PCR using a SYBR Premix Ex Taq kit (Vazyme) on a qTOWER3 real-time PCR machine. All quantitative primer information is shown in **Supplementary Table S1**. For each reported result, at least three independent biological samples were subjected to a minimum of three technical replicates. The results were normalized using the internal control *CaUBI-3*.

### 2.9 Statistical analysis

SPSS program version 19 (United States) was used to analyze the data. The one-way analysis of variance and *t*-test were used to determine the significant difference between groups.

## 3 Results

### 3.1 CaMYBA, CaMYC, and CaTTG1 form an MBW complex

Although the transcription factors CaMYBA, CaMYC, and CaTTG1 are expressed in the leaf of *C. annuum* L (Zhang et al., 2015; Lu et al., 2019), whether or not they regulate the anthocyanin pathway in pepper leaf as a complex remains to be confirmed. We compared CaMYBA and CaMYC with MYB and bHLH proteins that have been reported to regulate anthocyanin synthesis in other species. Similar to MYB proteins that regulate anthocyanin synthesis in other species, CaMYBA contains a highly conserved R2R3 domain in the N-terminal region, a bHLH-interaction motif and an ANDV motif in the R3 domain region, and a KPRPR[S/T]F motif in the C-terminal region (**Supplementary Figure S3**; Stracke et al., 2001; Yan et al., 2020). Similarly, CaMYC, like other bHLH proteins that regulate anthocyanin synthesis, has a MYB-interaction motif in the N-terminal and a conserved bHLH domain in the C-terminal (**Supplementary Figure S4**; Wang et al., 2019). CaTTG1 is highly similar to TTG1 protein in tomato with a typical WD40 protein domain, and it is also similar to TTG1 protein regulating anthocyanin synthesis in *Arabidopsis* and *Petunia* (Gonzalez et al., 2008; Albert et al., 2011; Wang et al., 2025).

**Figure 1:**
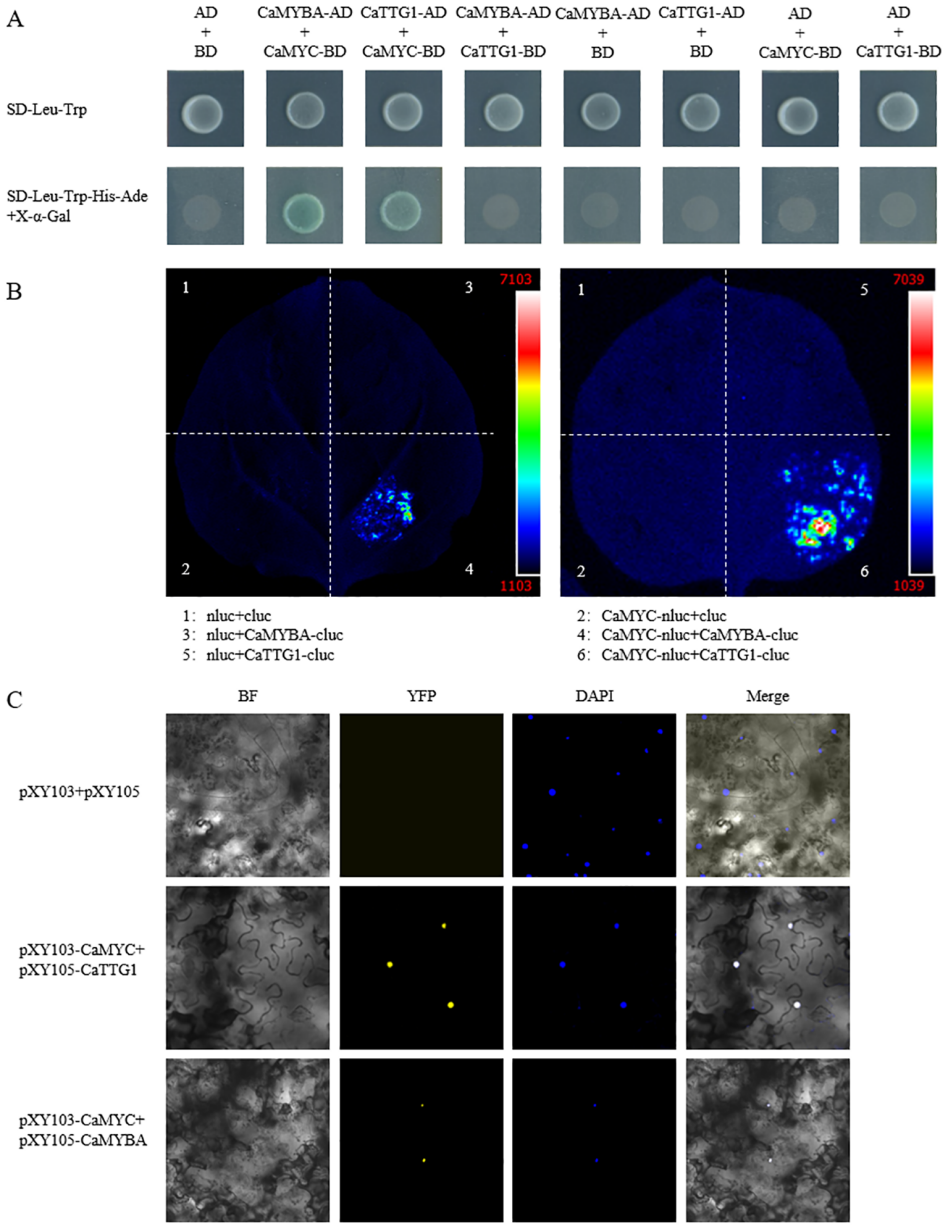
Interaction between *CaMYBA* and *CaMYC*, *CaMYC*, and CaTTG1. **(A)** Interaction of *CaMYBA* and *CaMYC*, *CaMYC*, and CaTTG1 in yeast two-hybrid (Y2H) assay. Transformed yeast cells were grown on SD-Leu-Trp and SD-Leu-Trp-His-Ade added with X-α-Gal. Transformation of pGADT7 (AD) and pGBKT7 (BD) vectors was used as a negative control. **(B)** *CaMYC* interacts with *CaMYBA* and CaTTG1 in the luciferase complementation assay (LCA). Positive luminescence observed by Charge Coupled Device (CCD) camera indicates mutual interaction. **(C)** Physical interactions of *CaMYBA* and *CaMYC*, *CaMYC*, and CaTTG1 by bimolecular fluorescence complementation (BiFC) assay. Transformation of pXY103 and pXY105 vectors was used as a negative control. Microscopic images were taken under bright field and fluorescence. Images under bright field (left one), YFP (left two), and DAPI (right two); the merged images (right one) are shown on the right. BF, bright field; YFP, yellow fluorescent protein.

To verify whether CaMYBA, CaMYC, and CaTTG1 can form a complex, we validated their pairwise interactions by Y2H assay. The results showed that only the yeast cells transformed with pGADT7-CaMYBA and pGBKT7-CaMYC, and pGADT7-CaTTG1 and pGBKT7-CaMYC could grow well and show GAL4 activity on the X-α-Gal-contained selective media (**Figure 1A**). In order to further verify the reliability of the interaction results of the Y2H assay, we conducted luciferase complementation assay (LCA) and bimolecular fluorescence complementation (BiFC) assay. The results of the LCA and BiFC assay confirmed the interaction results of the Y2H assay. Accordingly, CaMYC can interact with CaMYBA or CaTTG1 proteins, while CaMYBA cannot interact with CaTTG1 proteins, and they can form a CaMYBA–CaMYC–CaTTG1 MBW complex.

### 3.2 CaMYBA, CaMYC, and CaTTG1 synergistically regulate the anthocyanin pathway genes

**Figure 2:**
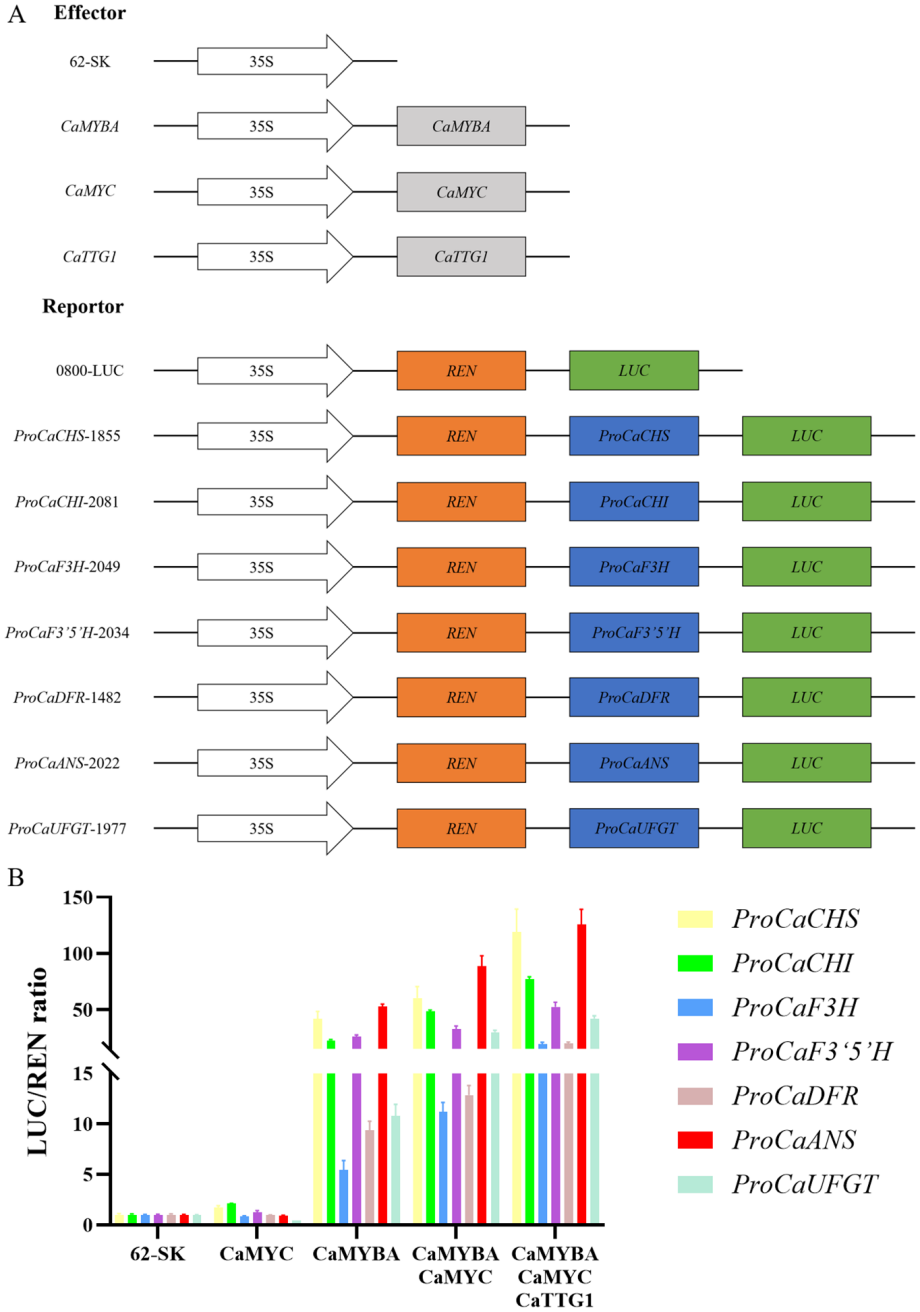
Collaborative regulation of CaMYBA, CaMYC, and CaTTG1 on the anthocyanin pathway genes of *Capsicum annuum* L. **(A)** Construct details for dual-luciferase assays. The effector constructs contain CaMYBA, CaMYC, and CaTTG1 driven by the CaMV 35 S promoter. The reporter constructs contain the firefly luciferase (LUC) driven by the promoter of *CaCHS*, *CaCHI*, *CaF3H*, *CaF3′5′H*, *CaDFR*, *CaANS*, or *CaUFGT*, and the Renilla luciferase (REN) driven by the CaMV 35 S promoter. **(B)** The effects of CaMYBA, CaMYC, and CaTTG1 individually and in combination on the promoter activity of *CaCHS*, *CaCHI*, *CaF3H*, *CaF3′5′H*, *CaDFR*, *CaANS*, or *CaUFGT* with the luciferase reporter assay. The 5′-non-coding regions are shown using colored bars for the seven genes tested. Empty effector vector (62-SK) was used as the control. LUC/REN ratio of the control (tobacco leaves co-transformed with the reporters and the empty effector vector) was taken as 1 for normalization. Error bars represent the mean ± SD of three biological replicates. Statistical significance was determined using Duncan’s t-test (p < 0.05).

To verify the regulatory effects of CaMYBA, CaMYC, and CaTTG1 on the transcriptional activation of structural genes in the anthocyanin synthesis pathway of pepper leaves, we collected their 5′-non-coding sequences upstream of the translation initiation site (**Supplementary Table S2**) to evaluate CaMYBA, CaMYC, and CaTTG1 regulatory capacity on the expression of each. The possible MYB-recognizing element (MRE) or bHLH-recognizing element (BRE) was predicted (Zhu et al., 2015), and it was found that there was at least one MRE and one BRE on the promoters of the seven anthocyanin synthesis pathway genes: *CaCHS*, *CaCHI*, *CaF3H*, *CaF3′5′H*, *CaDFR*, *CaANS*, and *CaUFGT* (**Supplementary Figure S5**). By single and combinatory tests of CaMYBA, CaMYC, and CaTTG1 as effectors in dual-luciferase assays (**Figure 2A**), we detected how they regulated the reporter gene via the 5′-non-coding regions of the pathway genes. The tests showed that CaMYBA could, alone, initiate transcription on the seven pathway gene promoters. By contrast, CaMYC alone failed to activate observable promoter activities in the same setting. When CaMYBA and CaMYC are together or when all three of them are together, greater promoter activity can be generated, and the presence of CaMYBA, CaMYC, and CaTTG1 at the same time produces the largest promoter activity (**Figure 2B**, **Supplementary Figure S6**). This suggests that CaMYBA, CaMYC, and CaTTG1 acted as an MBW complex in the transcriptional activation of the main anthocyanin pathway genes in pepper leaves, and *CaMYBA* should play a role as the main active factor.

### 3.3 Silencing of *CaMYBA* or *CaMYC* reduced the accumulation of anthocyanins and the expression of anthocyanin pathway genes

**Figure 3:**
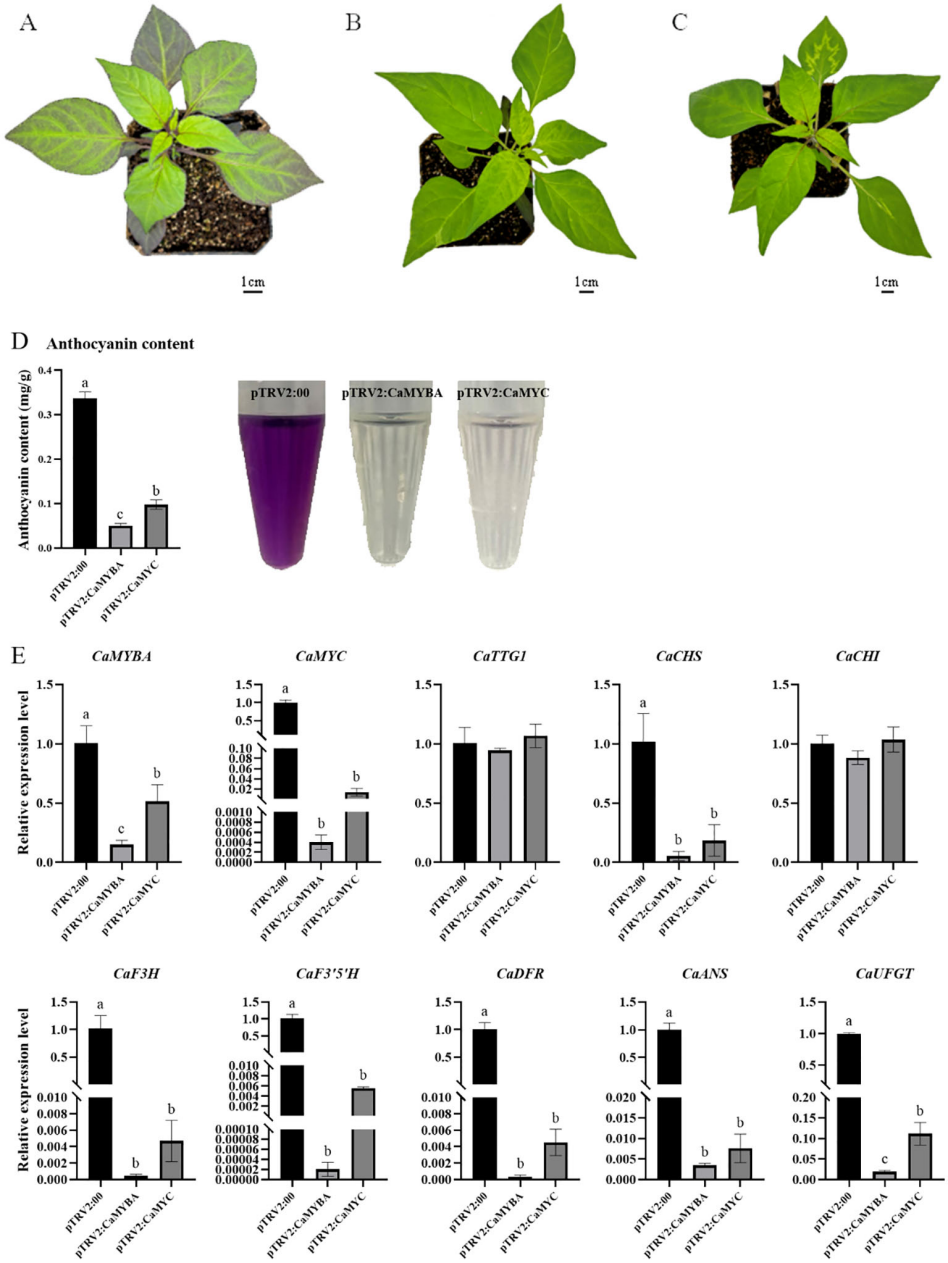
TRV-mediated silencing of CaMYBA or CaMYC in purple pepper plants (line PP). **(A)** pTRV2 empty vector infected pepper plant. **(B)** CaMYBA-silencing pepper plant. **(C)** CaMYC-silencing pepper plant. **(D)** Anthocyanin content in silencing plants. **(E)** Expression of anthocyanin synthesis pathway genes in silencing pepper plants. pTRV2:00, negative control plants; pTRV2:CaMYBA, CaMYBA-silencing plants; pTRV2:CaMYC, CaMYC-silencing plants. The expression of all genes was normalized by that of ubiquitin-conjugating protein gene *CaUBI-3*. The experiment was conducted with three biological replicates. Error bars represent the mean ± SD of three biological replicates. Statistical significance was determined using Duncan’s t-test (p < 0.05). Lower case letters indicate significant differences at the p<0.05 level.

To verify the role of *CaMYBA* or *CaMYC* in anthocyanin synthesis in pepper leaves, *CaMYBA* or *CaMYC* expression was silenced through the VIGS technique, in which the *Agrobacterium* strain harboring pTRV1 and pTRV2:00, pTRV2:CaMYBA, and pTRV2:CaMYC were injected into cotyledons of purple pepper line PP. After 5 weeks of injection, obvious green leaves were observed in the silenced peppers with the pTRV2:CaMYBA or pTRV2:CaMYC vector. After silencing *CaMYBA* or *CaMYC*, the purple pigment in pepper leaves obviously faded. However, no obvious symptoms were observed in pepper seedlings with pTRV2:00 (**Figures 3A–D**). The high silencing efficiency of *CaMYBA* or *CaMYC* gene expression after 5 weeks of injection suggested the reliability of the VIGS technique in silencing pepper gene expression (**Figure 3E**).

Real-time quantitative PCR analyses demonstrated that *CaMYBA* or *CaMYC* silencing did not impact *CaTTG1* and *CaCHI* expression. After the silencing of *CaMYBA*, the expression of *CaMYC* decreased significantly by as much as 99%. Similarly, after the silencing of *CaMYC*, the expression of *CaMYBA* also decreased to a certain extent by approximately 48%. The expression of *CaCHS*, *CaF3H*, *CaF3′5′H*, *CaDFR*, *CaANS*, and *CaUFGT* decreased significantly after *CaMYBA* silencing or *CaMYC* silencing was conducted due to low expression observed in silenced pepper leaves compared with the negative control. In contrast, the expression of these six genes decreased even more after the silencing of *CaMYBA* (**Figure 3E**).

### 3.4 *CaMYBA* in the MBW complex leads to activation of anthocyanin synthesis

**Figure 4:**
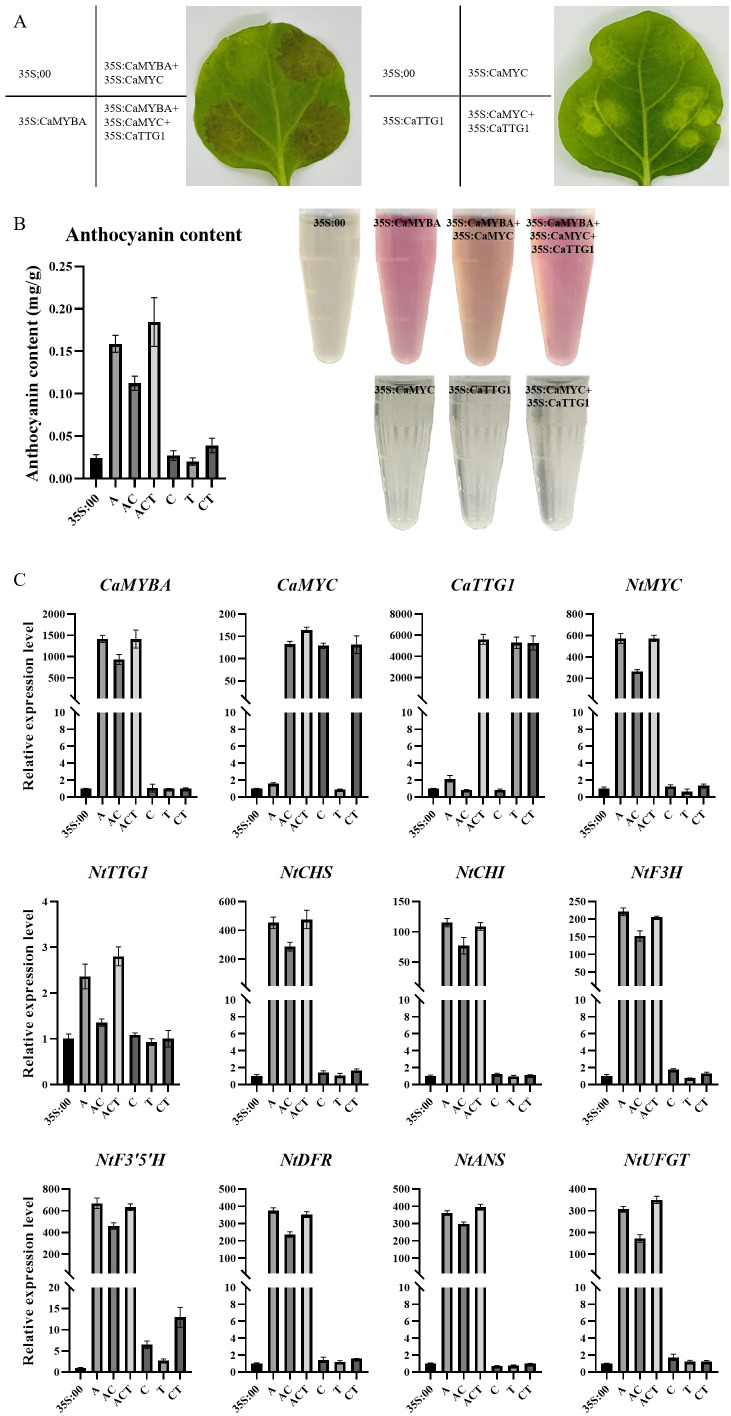
Transient overexpression phenotype in tobacco leaves. **(A)** Pigmentation of transiently overexpressed tobacco leaves in different combinations. **(B)** Determination of anthocyanin content in transiently overexpressed tobacco leaves in different combinations. 35S:00, negative control; A, 35S:CaMYBA; AC, 35S:CaMYBA+35S:CaMYC; ACT, 35S:CaMYBA+35S:CaMYC+35S:CaTTG1; C, 35S:CaMYC; T, 35S:CaTTG1; CT, 35S:CaMYC+CaTTG1. **(C)** qRT-PCR analysis of anthocyanin-related gene expression between tobacco leaves with different combinations of transient overexpression. *NtEF1α* was used as an internal control gene. Error bars represent the mean ± SD of three biological replicates. Statistical significance was determined using Duncan’s t-test (p < 0.05).

To confirm the role played by *CaMYBA*, *CaMYC*, and *CaTTG1* in the synthesis of anthocyanin, *Agrobacterium* carrying 35S:00, 35S:CaMYBA, 35S:CaMYC, or 35S:CaTTG1 was injected into tobacco leaves alone or in combination. Compared with the negative control, there were obvious color changes in the leaf regions with combined infection containing *CaMYBA* but no obvious color changes in the leaf regions without combined infection containing *CaMYBA* (**Figure 4A**). Consistent with observed phenotypic changes, total anthocyanin levels in tobacco leaf regions without *CaMYBA* overexpression were similar to those in negative controls, with no significant changes. In contrast, anthocyanin accumulation was much higher in leaf regions with *CaMYBA* overexpression (**Figure 4B**). In addition, no *NtMYBA* expression was detected in all overexpression combinations, so the effect of *NtMYBA* in tobacco leaves on anthocyanin accumulation could be excluded. The expression of *CaMYBA* was higher in tobacco leaf regions with color changes (**Figure 4C**). Therefore, ectopic transient overexpression of *CaMYBA* in tobacco leaves induces anthocyanin accumulation.

The effects of single or combined overexpression of *CaMYBA*, *CaMYC*, and *CaTTG1* on anthocyanin biosynthesis gene expression in tobacco leaves were analyzed by qRT-PCR. The CaMYBA-overexpressing, CaMYBA-CaMYC-overexpressing, and CaMYBA–CaMYC–CaTTG1-overexpressing regions presented remarkably higher expressions of *NtMYC*, *NtCHS*, *NtCHI*, *NtF3H*, *NtF3′5′H*, *NtDFR*, *NtANS*, and *NtUFGT* relative to negative control region and CaTTG1-overexpressing region, and this consistent with the greater content of anthocyanin in CaMYBA-overexpressing, CaMYBA-CaMYC-overexpressing, and CaMYBA–CaMYC–CaTTG1-overexpressing regions (**Figure 4C**). The expression levels of related genes did not change significantly in CaMYC-overexpressing, CaTTG1-overexpressing, and CaMYC-CaTTG1-overexpressing regions. Although the expression levels of *NtF3′5′H* in CaMYC-overexpressing and CaMYC-CaTTG1-overexpressing regions were higher than those in negative control, statistical analysis showed no significant difference (**Figure 4C**). Overall, *CaMYBA* dominated anthocyanin accumulation, and it may independently activate the expression of *NtMYC* in tobacco leaves and form a complex with it to further regulate anthocyanin synthesis.

### 3.5 CaMYBA binds to the *CaMYC* promoter and activates its transcription

**Figure 5:**
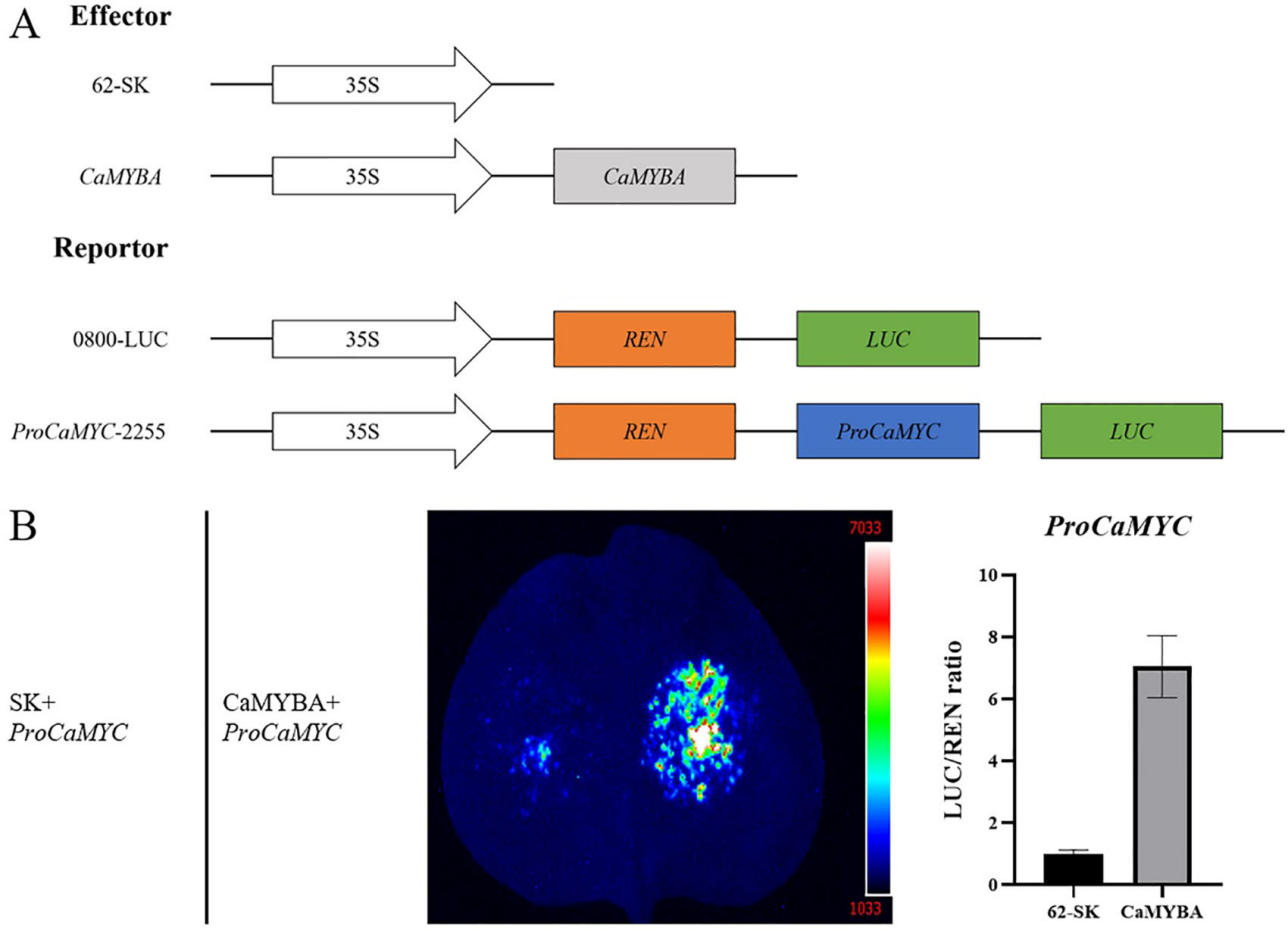
Regulation of CaMYBA on the *CaMYC*. **(A)** Construct details for dual-luciferase assays. The effector constructs contain CaMYBA driven by the CaMV35S promoter. The reporter constructs contain the firefly luciferase (LUC) driven by the promoter of *CaMYC* and the Renilla luciferase (REN) driven by the CaMV35S promoter. **(B)** Dual-luciferase detection experiments showed that CaMYBA promoted the expression of the *CaMYC*. Empty effector vector (62-SK) was used as the control. LUC/REN ratio of the control (tobacco leaves co-transformed with the reporters and the empty effector vector) was taken as 1 for normalization. Error bars represent the mean ± SD of three biological replicates. Statistical significance was determined using a t-test, p < 0.01 (**).

To verify the regulatory effects of *CaMYBA* on the transcriptional activation of CaMYC, we collected 5′-non-coding sequences of *CaMYC* upstream of the translation initiation site (**Supplementary Table S2**) to evaluate CaMYBA regulatory capacity on their expression (**Figure 5A**). Dual-luciferase assay showed that *CaMYBA* could initiate transcription on *CaMYC* promoters (**Figure 5B**). At the same time, we also performed transient overexpression of CaMYBA in pepper leaves. Although the phenotype change was not so obvious as in tobacco leaves, qRT-PCR data showed that the expression level of *CaMYBA* was significantly increased, and the expression levels of *CaMYC*, *CaCHS*, *CaCHI*, *CaANS*, and *CaUFGT* were also significantly increased (**Supplementary Figure S7**). These results all indicate that *CaMYBA* can activate the transcriptional expression of *CaMYC*.

## 4 Discussion

Chili pepper (*C. annuum* L.) originated in the tropics of South America, belongs to the Solanaceae family, and has high economic values (Wang et al., 2022). It has been reported that purple pepper leaves can enhance photosynthesis and alleviate oxidative stress (Dewez and Perreault, 2013; Zhang et al., 2015). In addition, pepper can also be used as an ornamental crop, and the purple pigment increases its ornamental value. Therefore, it is of great significance to expand the understanding of the regulatory mechanism of anthocyanin biosynthesis in pepper leaves.

In *Arabidopsis* and *Petunia*, MYB, bHLH, and WD40 transcription factors form an MBW complex to regulate anthocyanin biosynthesis (Gonzalez et al., 2008; Albert et al., 2011). According to previous reports, *CaMYBA*, *CaMYC*, and *CaTTG1* have been shown to be highly correlated with anthocyanin accumulation in pepper, and they may regulate anthocyanin biosynthesis in pepper (Borovsky et al., 2004; Aguilar-Barragán and Ochoa-Alejo, 2014; Zhang et al., 2015; Lu et al., 2019; Jung et al., 2019; Wang et al., 2025; Liu et al., 2024). However, whether they can form an MBW complex has not been reported. In this study, amino acid sequence analysis showed that CaMYBA and CaMYC were highly similar in structure to MYB and bHLH transcription factors regulating anthocyanin synthesis in other species (**Supplementary Figures S1** and **S2**); CaMYBA and CaMYC should be the corresponding members of the MBW complex regulating anthocyanin synthesis in capsicum. Meanwhile, protein interaction experiments showed that CaMYC could interact with CaMYBA or CaTTG1 (**Figures 1A–C**), and it was indicated that CaMYBA, CaMYC, and CaTTG1 can also form an MBW complex in pepper.

The binding of MYB and bHLH to promoters of anthocyanin structural genes was first reported with maize C1 and B (Roth et al., 1991). For the MYB part, C1 could bind to variable sites in the maize a1 gene promoter (Sainz et al., 1997). For the bHLH part, after the reported binding of CG-1 protein (Staiger et al., 1991) and human c-MYC (Blackwell et al., 1990) to CACGTG, the G-box was shown to bind to maize R (Kong et al., 2012), petunia AN1, and *Ipomoea* bHLH2 (Wang et al., 2013b). Most pathway genes identified so far do contain the *cis*-regulatory region with the necessary footings for the MBW complex; also, a 7-bp MRE (ANCNNCC) and a 6-bp bHLH-recognizing element [BRE and CACN(A/C/T)(G/T)] are required for an MBW complex to activate the promoter of target genes in the anthocyanin synthesis pathway (Zhu et al., 2015). After comparison, there was at least one MRE and one BRE on the promoter of *CaCHS*, *CaCHI*, *CaF3H*, *CaF3′5′H*, *CaDFR*, *CaANS*, and *CaUFGT* (**Supplementary Figure S5**). By single and combinatory tests of CaMYBA, CaMYC, and CaTTG1 as effectors in dual-luciferase assays (**Figure 2A**), CaMYBA, CaMYC, and CaTTG1 acted as an MBW complex in the transcriptional activation of the main anthocyanin pathway genes in pepper leaves (**Figure 2B**). Studies have shown that the activity of MYB transcription factors determines the function and regulatory mode of the MBW complex, with individual gene-family members regulating separate patterns (Davies et al., 2012), which act with common bHLH and WD40 transcription factors (Schwinn et al., 2006; Gonzalez et al., 2008; Albert et al., 2011; Lowry et al., 2012). Therefore, we believe that CaMYBA also determines the specific functional mode of an MBW complex regulating anthocyanin synthesis in pepper leaves. After VIGS silencing *CaMYBA* and *CaMYC* in our purple pepper variety PP, the leaves changed significantly from purple to green (**Figures 3A–C**), and the expression levels of anthocyanin synthesis structural genes (such as *CaCHS*) were significantly decreased except for *CaCHI*. In the study of Zhang et al. and Lu et al., the expression levels of all anthocyanin synthesis structure genes such as *CaCHS* were significantly decreased after silencing *CaMYBA* or *CaMYC* in pepper (Zhang et al., 2015; Lu et al., 2019). The difference is that the silencing of *CaMYC* resulted in a certain degree of decline in the expression of *CaMYBA* (**Figure 3B**). The VIGS experiment showed that *CaMYBA* and *CaMYC* have previously been shown to play a crucial role in the process of anthocyanin accumulation in pepper leaves. The transient overexpression of *CaMYBA*, *CaMYC*, and *CaTTG1* alone or in combination in tobacco leaves produced obvious anthocyanin accumulation only when *CaMYBA* was present (**Figures 4A, B**). Overall, CaMYBA determined the function and regulatory mode of CaMYBA–CaMYC–CaTTG1 complex to activate anthocyanin synthesis in pepper leaves.

In *Arabidopsis*, the bHLH transcription factor TRANSPARENT TESTA8 (AtTT8) regulates its own expression through an MBW complex, which ultimately contributes to the regulation of anthocyanin and proanthocyanidin (PA) synthesis (Baudry et al., 2006; Xu et al., 2013). In *Petunia*, ectopic expression of *PhAN2* (R2R3-MYB activator) in leaves resulted in ectopic expression of the bHLH transcription factor PhAN1 (Spelt et al., 2000). Similarly, *PhAN1* transcript levels are severely reduced in the anthers of petunias that lack a functional *PhAN4* allele (R2R3-MYB activator) (Spelt et al., 2000). After the expression of *CaMYBA* was silenced in pepper leaves, the expression of *CaMYC* was also decreased, and the expression of *NtMYC* was also significantly increased after transient overexpression of *CaMYBA* in tobacco leaves (**Figures 3D**, **4C**). This suggests that *CaMYC* may also be regulated by *CaMYBA* in pepper. In the promoter analysis of *CaMYC*, we also found several possible MRE and BRE sites (**Supplementary Figure S5**). Dual-luciferase assay also proved that CaMYBA binds to the *CaMYC* promoter and activates its transcription (**Figure 5B**). Transient overexpression of *CaMYBA* in pepper leaves showed no significant changes in leaf color phenotype, but the expression levels of *CaMYC* and some anthocyanin synthesis structural genes increased significantly (**Supplementary Figure S7**). Therefore, when combined with studies of bHLH transcription factors such as AtTT8 and PhAN1 in other plants, we speculated that the transcription of CaMYBA activated the expression of *CaMYC* in pepper leaves, and they formed an MBW complex together with CaTTG1 to further transcriptively activate the expression of anthocyanin synthesis structural genes such as *CaANS*, thus promoting the accumulation of anthocyanins in pepper leaves. Our results verified the formation of CaMYBA–CaMYC–CaTTG1 complex in pepper and verified its function and regulation mode in the anthocyanin synthesis of pepper leaves. However, it is not clear whether CaMYBA can stably activate anthocyanin synthesis in the absence of CaMYC. Further studies should focus on elucidating the effect of the presence of CaMYC and CaTTG1 on the functional stability of CaMYBA at the protein level and the validation of MRE and BRE on the promoter of the structural gene of anthocyanin synthesis in pepper. The verification of the CaMYBA–CaMYC–CaTTG1 complex and its function and regulatory mode will help to expand the understanding of the regulatory mechanism of anthocyanin synthesis in pepper leaves, improve the regulatory model of anthocyanin synthesis in pepper, and also help to create germplasm resources of pepper with different color patterns.

## 5 Conclusion

In sum, CaMYBA, CaMYC, and CaTTG1 can form an MBW complex, and the complex can directly bind promoters of anthocyanin synthesis structural genes such as *CaANS* to promote their transcription and expression, thus promoting anthocyanin accumulation in pepper leaves. We believe that CaMYBA activates *CaMYC* expression and determines the function and regulation mode of the MBW complex.

## Data Availability

The original contributions presented in the study are included in the article/**Supplementary Material**. Further inquiries can be directed to the corresponding author.
