# Supplementary material for: CaMYBA–CaMYC–CaTTG1 complex activates the transcription of anthocyanin synthesis structural genes and regulates anthocyanin accumulation in pepper (Capsicum annuum L.) leaves
Source: Front Plant Sci. 2025 Mar 7;16:1538607. doi: 10.3389/fpls.2025.1538607 (PMC11927005; doi:10.3389/fpls.2025.1538607)
Supplement: Supplementary file 1 [file DataSheet1.docx]

**Supplementary Table S1.** List of primers.

| Experiment | Gene | Primer name | Sequense(5’-3’) |
| --- | --- | --- | --- |
| VIGS | *CaMYBA* | pTRV2-CaMYBA-F | AgaaggcctccatggggatccACTAGACAAAGACGAACGCGAC |
|  |  | pTRV2-CaMYBA-R | GagacgcgtgagctcggtaccAGAAAAGTCATCCCAACCATCAC |
|  | *CaMYC* | pTRV2-CaMYC-F | AgaaggcctccatggggatccCGCATTCACATTATGGCGCA |
|  |  | pTRV2-CaMYC-R | GagacgcgtgagctcggtaccTCCGCAAGTACATGGTTCCC |
| Yeast two-hybrid | *CaMYBA* | pGADT7-CaMYBA-F | GccatggaggccagtgaattcATGAATACTGCTATTATTGCCAAGTCC |
|  |  | pGADT7-CaMYBA-R | CagctcgagctcgatggatccCTAATTAAGTAGATTCCATAGGTCAATATCA |
|  | *CaMYC* | pGBKT7-CaMYC-F | AtggccatggaggccgaattcATGGAGATCATACAGCCTAGCAGC |
|  |  | pGBKT7-CaMYC-R | AtgcggccgctgcaggtcgacCTAAGCGTCAAAAAATATACCTGCA |
|  | *CaTTG1* | pGADT7-CaTTG1-F | GccatggaggccagtgaattcATGGAAAATTCAAGCCAAG |
|  |  | pGADT7-CaTTG1-R | CagctcgagctcgatggatccTTATACTTTAAGCAGCTGC |
|  |  | pGBKT7-CaTTG1-F | AtggccatggaggccgaattcATGGAAAATTCAAGCCAAG |
|  |  | pGBKT7-CaTTG1-R | AtgcggccgctgcaggtcgacTTATACTTTAAGCAGCTGC |
| Luci-ferase complementation | *CaMYBA* | cLUC-MYBA-F | TacgcgtcccggggcggtaccATGAATACTGCTATTATTGCCAAGTCC |
|  |  | cLUC-MYBA-R | AcgaaagctctgcaggtcgacCTAATTAAGTAGATTCCATAGGTCAATATCA |
|  | *CaMYC* | nLUC-MYC-F | AcgggggacgagctcggtaccATGGAGATCATACAGCCTAGCAGC |
|  |  | nLUC-MYC-R | CgcgtacgagatctggtcgacAGCGTCAAAAAATATACCTGCATC |
|  | *CaTTG1* | cLUC-TTG1-F | TcccggggcggtaccATGGAAAATTCAAGCCAAG |
|  |  | cLUC-TTG1-R | AgctctgcaggtcgacTTATACTTTAAGCAGCTGC |
| Bimolecular fluorescence complementation | *CaMYBA* | pXY105-MYBA-F | AaggccggcggatccATGAATACTGCTATTATTGCCA |
|  |  | pXY105-MYBA-R | AgctctgcaggtcgacCTAATTAAGTAGATTCCATAGGT |
|  | *CaMYC* | pXY103-MYC-F | AggtacccggggatccATGGAGATCATACAGCCTA |
|  |  | pXY103-MYC-R | AccataccgccgtcgacAGCGTCAAAAAATATACCT |
|  | *CaTTG1* | pXY105-TTG1-F | AaggccggcggatccATGGAAAATTCAAGCCAAG |
|  |  | pXY105-TTG1-R | AgctctgcaggtcgacTTATACTTTAAGCAGCTGCAAC |
| Dual luciferase | *CaMYBA* | 62-SK-MYBA-F | AgaactagtggatccATGAATACTGCTATTATTGCCAAGT |
|  |  | 62-SK-MYBA-R | AcggtatcgataagcttCTAATTAAGTAGATTCCATAGGTCAA |
|  | *CaMYC* | 62-SK-MYC-F | AgaactagtggatccATGGAGATCATACAGCCTA |
|  |  | 62-SK-MYC-R | AcggtatcgataagcttCTAAGCGTCAAAAAATATACCTGCA |
|  | *CaTTG1* | 62-SK-CaTTG1-F | AgaactagtggatccATGGAAAATTCAAGCCAAG |
|  |  | 62-SK-CaTTG1-R | AcggtatcgataagcttTTATACTTTAAGCAGCTGC |
|  | *CaCHS* | 0800-LUC-CHS-F | acggtatcgataagcttGTGTTCCGATGGTTATGTCT |
|  |  | 0800-LUC-CHS-R2 | tggcgtcttccatggAGAAATAGCAACTAGCTGGT |
|  | *CaCHI* | 0800-LUC-CHI.N-F | AcggtatcgataagcttTGAATCCGAGAGTGGTCTTCA |
|  |  | 0800-LUC-CHI.N-R | TggcgtcttccatggATGAGGGTGTGTATTATAGGA |
|  | *CaF3H* | 0800-LUC-F3H.N-F | acggtatcgataagcttTCACGTGATTTGTCCTAACCAG |
|  |  | 0800-LUC-F3H.N-R | tggcgtcttccatggATGTTTGGGAGGGAGAATGGT |
|  | *CaF3’5’H* | 0800-LUC-F3’5’H.N-F | acggtatcgataagcttATCTGCTATCCCTTCATTATGG |
|  |  | 0800-LUC-F3’5’H.N-R | tggcgtcttccatggTGACTTAGGGATTTGGTAGGT |
|  | *CaDFR* | 0800-LUC-DFR-F | acggtatcgataagcttTGCTCTAAACATGACTGTCTTTAAT |
|  |  | 0800-LUC-DFR-R | ttggcgtcttccatggTTTCAAGGTAAAAGAGTAGGAAAAT |
|  | *CaANS* | 0800-LUC-ANS-F | acggtatcgataagcttTAACTTTTAATAGTTATGTAGGACTTTCT |
|  |  | 0800-LUC-ANS-R | tggcgtcttccatggCTCTGTAAGGTAGATTGAACACCA |
|  | *CaUFGT* | 0800-LUC-UFGT-F | acggtatcgataagcttAGTGTAGGTACCCGTACGATCC |
|  |  | 0800-LUC-UFGT-R | tggcgtcttccatggTCTTTTGTTGTTGAGTTTGTTTAAGT |
| QPCR | *CaUBI-3* | qCaUBI-3-F | TGTCCATCTGCTCTCTGTTG |
|  |  | qCaUBI-3-R | CACCCCAAGCACAATAAGAC |
|  | *CaMYBA* | qCaMYBA-F | ACGTACTAAGACCTCGCCCT |
|  |  | qCaMYBA-R | TGTCGCGTTCGTCTTTGTCT |
|  | *CaMYC* | qCaMYC-F | AGCCGTTCTTGAACACCCAT |
|  |  | qCaMYC-R | TGCGCCATAATGTGAATGCG |
|  | *CaTTG1* | qCaTTG1-F | AGGCATCAAGCGAGTGTGAA |
|  |  | qCaTTG1-R | CAAATAAGCGCCTGCCCATC |
|  | *CaCHS* | qCaCHS-F | CATTGGGGATTTCTGATTGG |
|  |  | qCaCHS-R | GGCCTTTCTCATTTCATCCA |
|  | *CaCHI* | qCaCHI-F | CCTTGCTGGTGCAGGGATTA |
|  |  | qCaCHI-R | GGAACGGCACTCTCTTCCAT |
|  | *CaF3H* | qCaF3H-F | ACCCTTGGGTTGAAAAGGCA |
|  |  | qCaF3H-R | TCTTGAACCTCCCGTTGCTC |
|  | *CaF3’5’H* | qCaF3’5’H-F | TGGCCTACAATGCCCAAGAC |
|  |  | qCaF3’5’H-R | ATATCCGCCACCACAACGC |
|  | *CaDFR* | qCaDFR-F | GAGACTTGCCGACAGAAGCA |
|  |  | qCaDFR-R | CTCCTTGCCACTTGCATAGTTT |
|  | *CaANS* | qCaANS-F | CAAATGCCCACAACCAGAACTAGC |
|  |  | qCaANS-R | CGCACTTTGCAGTTACCCACTTTC |
|  | *CaUFGT* | qCaUFGT-F | TGGTAGCCATGCAGTTCCTTT |
|  |  | qCaUFGT-R | GGGGTGTCATTGCCTTGTTT |
|  | *NtEF1α* | qNtEF1α-F | TGGTTGTGACTTTTGGTCCCA |
|  |  | qNtEF1α-F | ACAAACCCACGCTTGAGATCC |
|  | *NtMYBA* | qNtMYBA-F | CGGCCTCGAACCTTCTCAAA |
|  |  | qNtMYBA-R | AGCTAGTAAATCGGCCCACC |
|  | *NtMYC* | qNtMYC-F | ACGACGGAAAATGAGGAGGT |
|  |  | qNtMYC-R | TCAGCCAAGAAGACGCCATT |
|  | *NtTTG1* | qNtTTG1-F | ATGCGATTGCGTGGGCTCCACA |
|  |  | qNtTTG1-R | CAAGCAATCCAAAAGGGTTGCA |
|  | *NtCHS* | qNtCHS-F | AAGCAAGAGAAACTAAAGGCTACAAG |
|  |  | qNtCHS-R | AAATCCAAAAAGCACACCCCAT |
|  | *NtCHI* | qNtCHI-F | CGGGTGCCTCCATTCTTTTTACT |
|  |  | qNtCHI-R | CCTGACACTCTTTCGGCGATACTAC |
|  | *NtF3H* | qNtF3H-F | CCAGACAAACCAGATGGATGGATAG |
|  |  | qNtF3H-R | CAAGGGTAAGGTCGGGCTGTG |
|  | *NtF3’5’H* | qNtF3’5’H-F | CGCACTACCATACTTAGGAGCCAT |
|  |  | qNtF3’5’H-R | CAGCATCAGGAGTAGAAGCAACAG |
|  | *NtDFR* | qNtDFR-F | GCAGTTGCTTCCCTTTTCTACC |
|  |  | qNtDFR-R | TTCCCCATTGGTTGACTTTCC |
|  | *NtANS* | qNtANS-F | GTGCCTGGGTTACAACTTTTCTATG |
|  |  | qNtANS-R | CATTGCTTAGGATTTCAAGGGTGTC |
|  | *NtUFGT* | qNtUFGT-F | GAGTGCATTGGATGCCTTTT |
|  |  | qNtUFGT-R | CCAGCTCCATTAGGTCCTTG |
| The DNA restrcition enzyme cutting sites are underlined. | | | |

**Supplementary Table S2.** Accessions of genes in this study.

| Gene | ID |
| --- | --- |
| *CaMYBA* | Capana10g001433 |
| *CaMYC* | Capana09g001426 |
| *CaTTG1* | Capana03g001813 |
| *CaUBI-3* | LOC107873556 |
| *CaCHS* | LOC107871256 |
| *CaCHI* | LOC107852750 |
| *CaF3H* | LOC107859880 |
| *CaF3’5’H* | LOC107848667 |
| *CaDFR* | LOC107860031 |
| *CaANS* | LOC107866341 |
| *CaUFGT* | LOC107843659 |


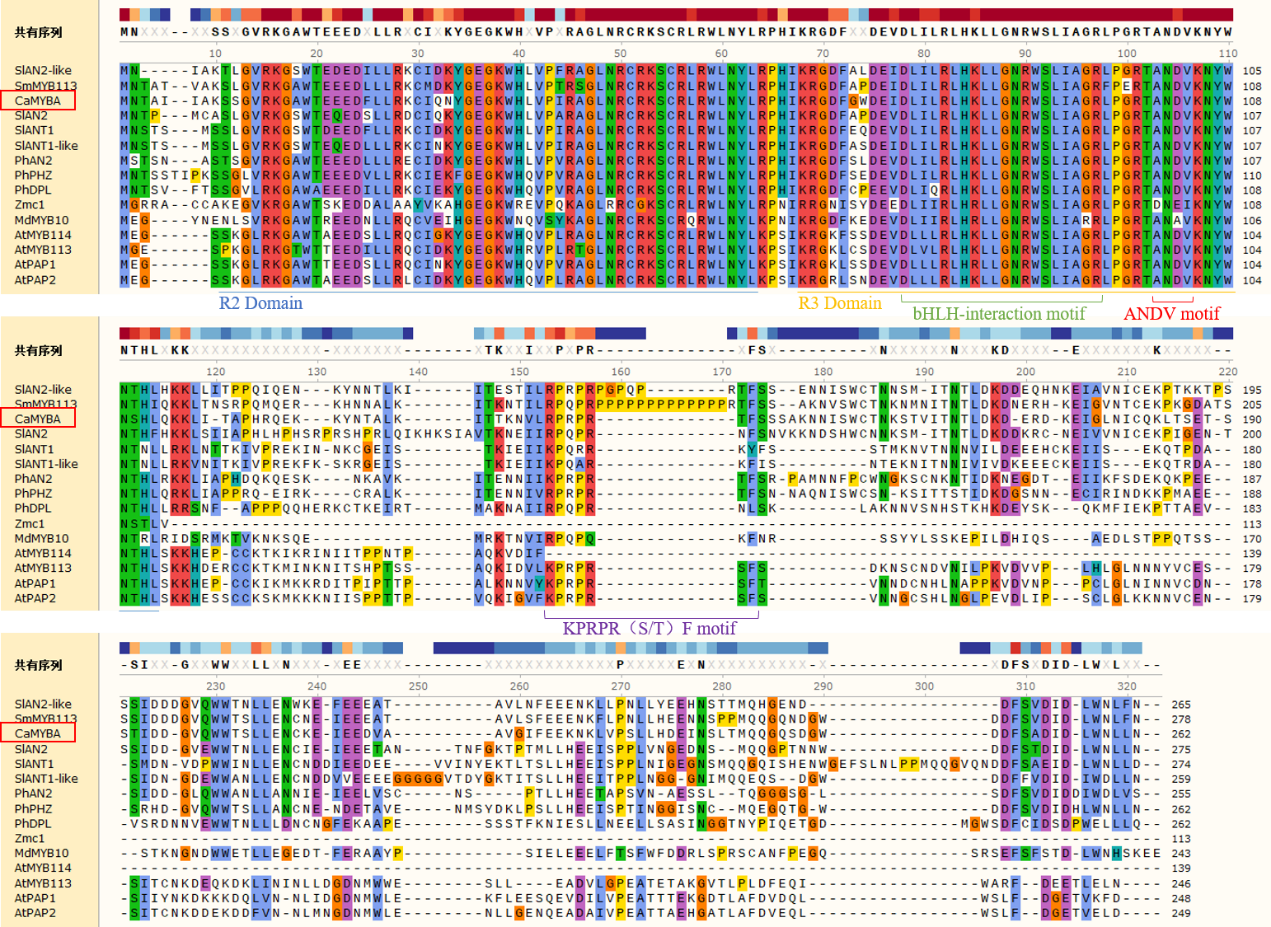


**Supplementary Fig. S3.** Amino acid sequence alignment of CaMYBA with MYB transcription factors regulating anthocyanin synthesis in other species. SlAN2-like: NP_001362565.1, SmMYB113: AGK37072.1, SlAN2: NP_001265992.1, SlANT1: NP_001234417.1, SlANT1-like: XP_004249668.1, PhAN2: AAF66727.1, PhPHZ: ADW94951.1, PhDPL: ADW94950.1, ZmC1: AAK81903.1, MdMYB10: XP_028963316.1, AtMYB114: NP_176812.1, AtMYB113: NP_176811.1, AtPAP1: NP_176057.1, AtPAP2: NP_176813.1.


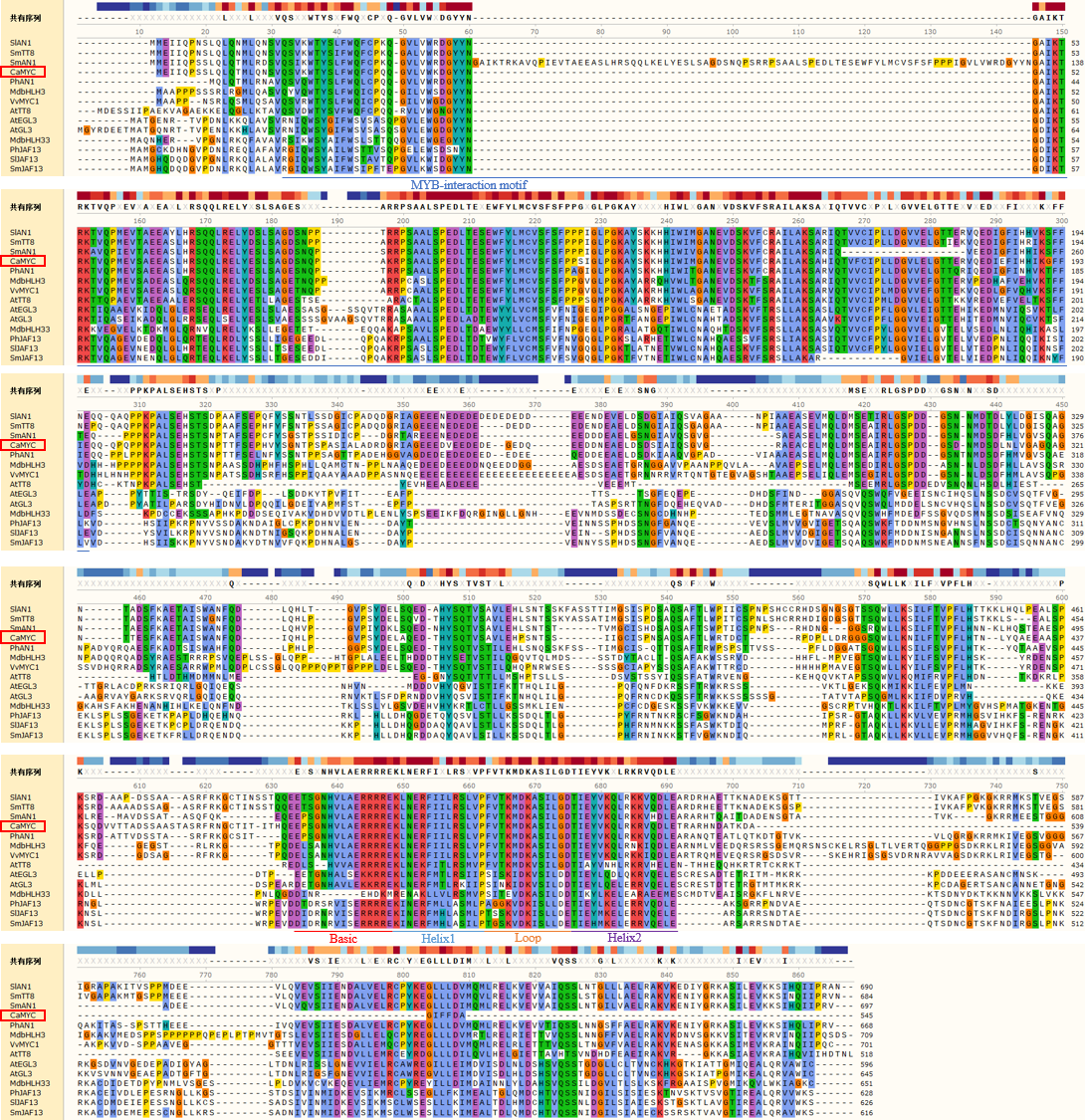


**Supplementary Fig. S4.** Amino acid sequence alignment of CaMYC with bHLH transcription factors regulating anthocyanin synthesis in other species. SlAN1: XP_049408052.1, SmTT8: XP_015086121.1, SmAN1: AMJ52090.1, PhAN1: AAG25927.1, MdbHLH3: ADL36597.1, VvMYC1: NP_001268182.1, AtTT8: NP_192720.2, AtEGL3: NP_001185302.1, AtGL3: NP_001332706.1, MdbHLH33: ABB84474.1, PhJAF13: AAC39455.1, SlJAF13: NP_001333930.1, SmJAF13 AIP93872.1.

>*ProCaCHS*

gtgttccgatggttatgtcttaattggatatttcaacactacttttttcatctcaaattatttgttgtgattttaaaaacagtctcaaattatttgtcttgttagaagttcataataaaattgattattttttaattttatttttcatagtaattattcttgaagactagaaatacaaaagtggaagaaatatttaataaagagagattatattttataacataaacaaaggtaagaacttcaaaaactcttgctaattaatatttttttataaatgtgtaaaagagaaacattataaataatatgaaatgtaggaactatatcggaaaatgtttgtattagtatatccaagtttagaataatttaaaggtactttcttatttaaccaaaactagaacattctattatatacgtcttgtactcttgttgtgaatgtttattttccttatcataactaaaaatgattttaaaaagtaaaaataaatccatttggaatgaatttattgaccaaaaaacaatgacgtatcgagtttgagaagttggtagcgttagtggtggatggagcttgtagataaaactattatttcctccgtcagataatacttgtccatattattaaaaatagatatttgataatatttgtttaatttaagaagttaacagctgatttatcttttttctacttattttaccattggttattaattatagtcatcactcaatacatttttcaaagcattgaataaactagaattacccatattatttattatttcttaatatgtgacgtcaaatcaatagtagacaagtattattgaacgaagaaagttctacgaaattcagtatatttatttctagagtttaagttatagacgttgtcaatgaaaaaaaagttttgcaccaccagatcattttttaactattataatagattatttatcttattttaagattacatatcatacatttatatacctgtaaatatctttcgggcatgatctaacaatgcaaaaaaaattttttacgcagtgacggaactctttttatatatatatatatatatatatatatatatattgatatttcttaataaaattcttgactctgtcattgatactaacgatgtatttaacttcaatattatttttctcgttaatgcatcctagctataagattttaaatattgaaaacaacaaaattaaattctcaattcgacgaaaaacctccaacttgttttgaaggttaattgcttagaagttggaaaatgaaagttgggacaaaagtagcaaaaaaataataaaaaaggaaaggggtggggaggtggaaccaaaattttaattcaaaattctaagaagccaacataggaagaagtcatatggaatttaacatctactatataaacatacaaaaaataattttaataatatatcaatggtgtaatttttcattgaagggattcggatgaacccctcggccctaggggcggagccacctttagctttgggggttcatcaaaactcccttcgacagaaaattatacaatttatatatgatttaaattattttatgtatatatagatgactttgaattttcttcgattaattcgtatgtttatttttgaatcccttagtgaatattctgaatccaccactgcgctcgatcctacttgattgtgggtggatgggggttggggtgaggagggggtggtggtggtggtgaggtgatatcatgtgccaaccaagcccaaaaaagttgcccccccacgtgaatactaactaccagtttgttataaaagtttcttcttataaatagccaaaccccatgcaaactataagtgcactacaacacacctca**ATG**

>*ProCaCHI*

tgaatccgagagtggtcttcaaaagggtcctcgattctttgaattagtggactatttttgaagtgtgtttggactattttgagccaattccgtgtgttccttgattttcttatataaggtacggtgcctgcattggaaccccgactaatccctccaacacacttttttttggtttcccatccagtgtccggtgctcgtattagagccccgattaatctgaatcgcgcgttgcagctcccaacagagttttctctatacccagggtcaaaccctcgacctctggtagacaatccattcactgcaccacaatccatttggtcctccaatacacttgttgttataggacacttgtcacttgtaaaatcaaatggttcaaattactccatttgcttttacttatcacatttttttactgacatagcatggtaagtgtaacacactcaaaagtcaaacggagcgtgtgaagtttcttaattagctgaattttttttaaaattatgctttgacacactcattaagaaaaataattaataacataactattttatcacaatactcttattaaatgatgcttacattatgtcttgaaattaatttgaagaaaaaatattaatgttaagagtaaaatagaaaaaaaagattatatgccaaaaatgataagtaaaaataaaatatataatttaaagattaataacagataaagtgaacggagggagtacttctcctcactaatcactttccaaacataacaacctttcttttgccttgttcatgctcaaaaagggacatgtgaagtgattttacaagtgaaatgtatccatataaaaaagggatatgtatgcatagtattaattaggaatgttaattactaccttaaatagcagagaaaataacaatgaaagaaaaaggggtatgtattaattaggatgtgaattaattctttaattagtgctccttcactacttttaaaatgagagtaaaaaggaaaaattctttttcatttttgggataaacaatgaatttcttgtggatcagattacatatcaactcaattttaaaaggaattgaaatttaatagatcaaaatgaactaaattactttttccgtgttcgcttttacttgttacaaatttcttaattgaatttttacttttacttgtcatcttttacatatcaagagtagacaactttttttattttttattttattttacccttaacattaatttttttttttcaaattaatttcaagatacacaatgtaaacatcatttaataggggtactacggtaaaataactatgttattaattatttttcttaataggtgtgtcaattttaaaatgtgataagtaaaaccaaacgggggagtagcatgattactttatcatagtacctctattaaatgttgtttacattgtgtcttgaaattatttttggagaaaaaataattaacgctaagggtaaaataaaaaaaaaaaggtgtcttctctttatatgtcaaaaatgacaagtaaaaatacaaatttaattaaaaaaattagtgacaagtaaaagcaaactgaggaagtataatggttgaattatgctgaatcatactcgtaaaagcaaactgaggaagtataatggttgaattgtgctgaatcatactcttatgaattgatttgttacttctgagtctaggaacgcattatgaacaagtaaagtgaacaaatggagtatattgaaatgggaaaattataatttaacttttaaagattgaaaaattatataagttaataactaaatttgggttgggtggggtggggggtgggaatggggggtggggggtgggggtgggcgtgggggtgggggtgagggtggggtggtaagcaaggaaagttgggtaaggcctttagctgccaaggtagaggtttttgactagtgacacattgtggtgagtaataactgtcaactgtgagcctcaagctctacctaactctaaattttcatggtcaattctacccttcaaaccactccattattaccattaaaccattatctcccctctatttaatctttttcctataatacacaccctcat**ATG**

>Pro*CaF3H*

tcacgtgatttgtcctaaccagcattctttgaattttccatattactaacaaaaaaagttagacaagtctttttaatctaaaagatactacgtgggtaaatcttctcaaaatgttaaatttgtaatttcaaaatataaataaagaaattactatatataataagagggagtgttgaggagctttccgtcatccttctaatcgatatgaggtattttaggcattgtgctttgctactgatgtatttgttggttttaagtaacttttatattatatttttggactaaaatatcaggacattttgtcataatttcattaaagatgttcccaatttttcttagtaatgcgtcccacttcaatattcatttcgcatttcagtgacttcgtcgctatttttagacctaccaatgaacgttcttttacatttctgataaactaagtaatagtactactcctgtaatttactactactatgatttactccatttcaattagatgtaaattaatatgtactgctcttattgttttagtttattttccattttttttactatattttgaaagttagttaaaagtactactttctccgtttaaaaaagaaggatctactttcctttttagtcaatttaaaaagaatgattcctttccttttttggcaatactttaatttcaactttccacgtagcatgtttatgaccacaagattaaaggacattttggtacattagacaatactttaatttaaggacacaagattcaaaagttctttattttcttaaactttgagtcaaatcaaagtaggttattctttttgaaacggagggagtatatatcataaaagaaacgtatacaaaaatagattcgctataaaaattttataactgtcacataaattgagataaaaagagtaatatatagtagtatttaaaagttaagtcaaaaaatcataaattacgataaataacaatttaaaatatttaaaatcatatatataaaattttaattgactctccaaatttcatttatgtcataaatagacattgtgataataaaagaaataacatatataaattgaaatgatataaaaaatactataaattgagacagataaaataacattgcacggacctcatatctacttacctatataaaagacaaataaatctgacgatataaaaaattcttatatgatatactatatttaaatttaaattgattaattttatattcgatatggatgggaaaacaacaaagcagctttttggttgcgtttgtttttcttaaacacaaaattaaataaataatgttttaattttttaaattttattcttaaataaatagtgttttagattatctagaaaacattaataaaaagattcttttatttttatccttagtagttttatttgattaagaaattatttttaaaaggacatttcatctttttaaacgtatgtattaagaataaattataaaaacactcattattcagcaatttataaaaggaggcataaaaccaaaaaaggcaatttaaagaagtgttatctagaacattattccctccttctcaaattatgtgtcaccattttctttttagtccgtttcaaaaaaaatgtcatctttttttatttgataaatttttaaagacacaattataatttcacccttggtgattccacttaattttaaatattattagtccttttttatttatcttttcaattaaaaatggtctcacttgattttaaatattattaatactttatttaagaagtaattttaataaagtcaatacctactttttaaatattatacccgatcaaatggcgatacataatttagaacggagtgaagtctttcgtattaagtactatttgccaacattggtatctacccttcaactctagaaagaaagcacgtgaaaagttggctgccacgagaggtatttaggtgtccacgttatcaatgaattcactcattccatatatttcagcccctctatatatttatttcccctatattcactacttaccattctccctcccaaacat**ATG**

>Pro*CaF3’5’H*

ATCTGCTATCCCTTCATTATGGGGACGGCTTAACGCATAAAGTTCTCCAGATTACTTGTACGATGCTGTTCTTTTTGGAACTGTGACTTTGCTGGTGTAATAATTACAAAAAAGTTGCTTCATTTTCATCGGCTTCGTCTTCGTCTTCATCTCCTCCTTTTTCGATTTTTGACATTTAACCTTTGCGAGGAACTGATTATGAGCAGTTATGAAGTCGAATACTAAATAGGATCGATTTCCTACATTTGGTATCCACAGGTCATGTATATGTCTGGTATGAAGGAAAATGATTTTCCACCATATACAGTAATCACATGATCATCACAAAAACAACCACATGCCTAGTCTAACCAACATGGGTTGTGGTGTAGTGGTGATACTACTTCACCCTTAATCAGAGGTATCGGGTTTGAGCCTTGGGTATGGAGAAAATCTTGTTGGGACCGCCAACCCCATAATGGGCTTTGCAGTGCACAATTCAGATTAGTCGAGGCTTCAATGAGAGTATCGGACATTGGGTGGAAAATAAAAAAAAAATTTGTCCAATATATTTCCACAAAATGAGGTCTGTAGAGGGTAAAGTACTCAGGTAGAAAGACTGTTTTCGATAGACTAGCAGCCTCAAGACAAAAGAGTTTAATGTAAGACCACATAGAAAGTAAAGTGTACAAAGTTTCGCAGTCCCTAGGTAGAAAGATCTTTTTTGATAAACCAGCCTCAAGACAAAAATACAGATGTGAAATAAGGCAATTTGTCCATTTAGGAATATGTTGTCAACCACCACGTGTATCTATATATATgatataaaatatagtcaaaattagagttggttaagacttagggtgcacattgcaatccaaccccctgctagtagggggttgagctattatatgtatatatttagtcaaacttttcgaggttatttgttgggatattttttgttgctatagtaaaagaattgtatagagaacattattttaaataaaatttgatcgttataataaagtgctgttacgaataataagataactgttatataaaatcagagacggatgtaattttgtcgaaaagtattatttgaagtcaatatttttgacataagacataaatttgtgtaaaaaatttactaatattataacaaatagtagattcgaactgataataccttaacgattaaacccatatataaattctgacacataattaggagtaaacttttaagtttgtggattacaaTGTAGAATTTGTTACTCCACCATTTGACTTAGCTCTTATAATATTATTATTTTTTATTTGATCATTTATATAAATTAAGAGAAACTTATAATTTTCCTCCTAAATTGTCATCATTGTTATGGTTTCAAAAATATCGTCATTATCATTAAATAACTACCTGAAGTATTACTCGCAATCAAATTTAAAATTTTAAAATATAATTAATACAATTAATTTAATAAAATAAACTTTTAATAAATATTTCTTTTAAAGAATGTCAAATCAAGTTATACATGTTCATATACATTACATATAGATTATATTTTACTTAATACATATATTATACATTCGCAACTATTTTTAATTGAAACGATTGGATGAATAACTATTCTGGTTGATTCTTCGAATATTTAATGGGAAAAATTCAGAAATAGTAACTCTATAGTCTTAATTATAACTTATATAGCAACAGTTTCATAATTATGAAAAATAACAAATTGTATTTGTATTCAAGTAAAATGTTGTTATATAAATACATATACAAATGTATATGTATTACTCATAAATACATATGCACAACTGAAAAATACATATTCTTCTATTACTATGAAGTGGGTAAAATATTGCTACTTTTGCTATAAGTTGTAATTTTAAAAAAGTGTTGTTATTTATTGTAATTATAATCTTAAGGATGCTAGTTTCTGTAATTTTTCGCAACAACAATCATTTTAAAGGCAGCTATACAATATGTTTGAATGGTAGATGCAAAAATTCAACGTGACATTCACTTACAAATATACCTACCAAATCCCTAAGTCA**ATG**

>*ProCaDFR*

TGCTCTAAACATGACTGTCTTTAATAAGTACGTACAAGTTACAAACAGTAAAAAAACTCCCAAATTTCTTGAAATATAGGCTTAAGTAAAATGAAGGAATAGATTACATTAAAGTGAAGAAGATATTGAATTAATGATGATGAAGAAAACAAATAAAGGATATGTTATATATACGGTACTTAGGTTGGGCAACTAGCTAGCTAAGAGACTTGGTATTTAATTCCAAATATTAGTTGTCAAAAGTTGCGTGCGCTATGTCTATGTGGTTTGTTTTAAGGGTGTTGAAATAAAGCAAGTATATATATGGCTAAGCAGAGCTGGAATTCCTTGTTTTGACTTCTCGTGCAAAATCAAATTAATTAATTTAATGAAAATCTTGACCATATCCGGCCAACAACAACAATATTAATCAGGGAAATGTGAATGGAGCTTCCAAGTTCCAATTAGTCATGCTATTAATTAAGAAAAATACTAGTAATATTTGGGCTGGTTCTGTCCATGATGACAAATTTTTATTTTTGTTTATGTATCTTTAGTTTTGTTGAAAACAAATCAATTTGGATATATTATTTGATTTGTAAAAGACAAACGTGTTTGAACCTTTACAATCCGTTTTTGAGAATGCAGGAAAAGTGATAGAAAGTTCGTTAGCTTATAAGTTATGATATAAGATATTATATTAAATTAAAGTTTTAACATAGATTATTGTCATCCCCTCTTTATGTTACACTTTTAGGTTTAAGGACCTGAATGAAGATTATAGTTTTCCTTATATAAAATGGAAGATAGCATAAAATTAATCTTATGATTCTTTTTGGAAAAAATAAACATGGTTTATTGTCCAAAATATAACAATATTGTCACTACTAATTGGTATAATTCTTATCAAATATTCTGCTCATTTTCTATTGAGGAGTCTTAATTATTAATAGACACATAAAAACAATTTTCTCTTCATTTGAAGGATTTGAAACCTTGTTACTTTAGGTGTAACAAACTTCCATATACAACAACAATATATACAATATCATCCCATAAATTGGGTCTAAAGAAGATAGAATATATAGCAATTTTACCCATTTTCGTGAGATAAAAAGATTGTTTCTAATAAATCTACAGACTTAAAAAAGTGAAATCAAAGGACCGTACAACTTAGTTCTGTATTATATGTAGAGAAGCAAGTGGCAGCCACATTATCCAGACAGTTCACACTAGCCCTCCCAAAGCCCAAAAGGCCACAACCACAACCCATTTTCTCACCAACCAGTCTTATTTGGCTAATCCAACCAAGTTACTCAAGGGTACGTTGAGAGCACGTGCTTACCATCTAACATTACTACTCCTAACACCACCTACATGCAGGCAGTATAAAAACAAAATACTACATAAACATAAATAATACCTCATCTCCTCGTAATTGTTATACTAGTAATTAAGTCCAGTCACTTGAAGATTTAATTTTCCTACTCTTTTACCTTGAAA**ATG**

>*ProCaANS*

TaacttttaatagttatgtaggactttctttattagtaaaattagaacaacattatcaatactttcttgattatttaaaacatcacttatttaagggtaaataattttaaaaaataattaaaacataaataaagagaatagtaaataggaaaaatagattaggcttgtgcacacaagagttgggctgcaacccacaaggcaaaatgggtcgatatcctcggtaaaaacttacacgcacccgatttatacaccaagctaatgatcgctaccacggttatgtaattaaattaagtaaaatatatattgattaatcatggttaagtaacataactttaaattcaaacgcatggcatgcatatattgatttgatgtatacaccgggtgcgtgtaagttttactccaatatcctccccgtgggagacAtgtttttgtttcttctattcttagttggattttattttttgaagttcaagtagatgatggtaaaaattacttttatcgatcttgatttgcatataaatttccgacttacggacctagctgaaccagagagcattttctcgctgttgagttctagaaatacacttaatcgcattatgattgtgtcatttaaatacaagataaacctgcttatattttcattgtggacaataggttgtccgactctttttcgaattgcgtggatagcgaaagcttagtgcatcgagctgctattataaaccattatgtaatagaaatgttagcttcttttttttgttttgtttttttggaaccccaaagaaaccaacaatctttgtcatagcctctgtccctcggttgatcactctatgctgAacattttgtgataacttgcttaccaaacaatagcttgcagaaatgttagcttcgatagttgcaataaagacggttctaagtcccagctatcagtacaggttctaaaataagaaatctcagttatttattgaagaacaaactatagtttttaaccacctttctcatgttaaaccacccttttttagtctttcaaaagtttgggttttaagttgttaagaaataagaaatgtcagttatttaatgaagaacaaactatagtttttaaccacttttttctcatgttgaaccactctattttaatcTttcaaaagtttgggttttaagttgtcaaagacttatttgttaaagaggcacatagttttggtgaaaatcatctattgaagggcatttataaccaattttttgctcaaaatgtttatttcttgatatgattctagcactcttattattattagttgcatgtaaagtgccttttccctgatgtttaagtattccttttcccctgatgtggaaaacttccgtggaatacctgctaccgtccactaacaaaaaacactgcgtaattctgttcaaagagaagaagaaatcacctacaagtagtatttttgcttgtactgaaattaaacccgagacctcgtgattttcaacacatttcattgatcactaatccgcaccctcagggtccaaatccacccttatcatcccacgtttagtggagtatcAattaaatgagatgtaactcgtatgatttagccagttaagtatctttttaaagtaatataaccaaactaaaataagtatgttaatggaaaacaacatagggagaaaacagggagctccagttcccagccaccagtcatttgtttccacatataacattatagaattgtgagccaaccaccattctaattttaggcAaaactaaagactgaaaaccttcatacaatatacaaaaaaggttagcataagtcacgtgcgtgcatgattgttgaatgctttaagctttatcttccagtcaaaataacagaacttacctactttggcatccacaatataaacctcaactagatgaccaaataaagggtaagaaagaaaaaaagcaagagaaggaattaatttgtagtcatatttcttggtacttctcttcatttttcgagattgggttggtgttcaatctaccttacagag**ATG**

>*ProCaUFGT*

AgtgtaggtacccgtacgatccgttagtgtgggtcgtgacaatatatatatatatatatatatatatagatagatagatagatagatagatagatagatagatagatagatatagtacttttttatttcataaagaataacctagtttgacttgacatgaaatttaagaaaataaagaaatttttgaatcttatgatcatAaattaaagttatgtcaattattataatattttttaattttataatcttaaatatatcacgtgaaaaattaaaattaaatataaccaaaaaaattattatTttaaataaattaaaaaactaaattattctttttaaaacggggagggtaatataattatttggatggagcaatcaaatacattaaaagctcaacttaattctaaaggcggaccataaagagtttgttcgggtgtcccgtttaacgatcaaaatttttacacgaaaaagaaaaatcaaagaaacattttttggaaaaaaaaAaatctgtggcagccaaagtttccacgtccaaaagagtacgcaatacgtagagccaatatctaccgtacattccagcttattatttatgttggatttttaAatttatagagcaaattaaattaataaaatttggatttttcaatttcgtacttttgatgttttcgaattttttgagtttttccaattttattcgataaagtcttcatataaccatataattaatttgtgcttcaaatatttttttacacctagcaagatacaataatcttacgtgttaaataacattaaatattaaaaaaaatttagttcatgtatttatacaactaaataatagatatccaacattattgttattcctagtgtttaattgaatctttttgtaagcattaatattgatttggattttatttgagttactaatatttattagttataaaacttattgaaccattcaaaattttaagtttaaacttgaaataatatgttgattaaaaaaatatgaaaaagtttaagaaatatttataaattatataataataaatatttttaaaaattatataaatataatattgaattagtttgattcgatttgacttattTtaattaaaatcaaagcaaattaattataatcgattcttttttaacatcaaatcaagttaaactaagccgttagtctttttttttttatttgactcgagtTacgatttgtataatttacaattcgattttgttcgccgtgtagctcacattaccgtagatcctgccaaactcaaatgcctggcacatcttccactctagtGaattccttgtgtaattatgcttctatgttgaataatgaaggtttctagtggacccatcgaatgtaggtaaggtaaatatttcttcgtatcgaacgtttaCaaaaaataacagcaataataacaaattagatttcattatgattgtaattatagaagtatgtaatcatcattttcgcaccactgatttgtaaagtcgaagaagcaactcttttttctttagaaacatggctaaatattgaagactattaaatactatcataagtgacatattcagtataaaatcataaatgaaatatgaattaatttaaaaaactgaacatatggactacttggccgtagagaaatattgaacttctaaattatattattcccactctatacatttggcattaaatatgtcaaggatcaatgataataacggctcacactaatcgagagatgaattgtaatcatcttgagtttaaattatgagcagaatccattacttttgcatccacctttcaagaaaccaattaatcacatgcatcaacccccaaaatggttggttttatcttcaactaacccccaacaagcaaagtacaaccaccaaccatttatctaccacacaatcccaagtgaacccacaacaaactataaaaaggtcaactttcacttaaacaaactcaacaacaaaaga**ATG**

>*ProCaMYC*

aatttcacatattattagaatttttttgtgaatttgagacttgattaataagtagtaactttatgtaatattataaatatttatgtaattaacattttaaaatttaaattaagattataaaaataatatattttaaaaagtgggttggcccgtgaagtcccacggcgcataatatgatgggttgggcttgctattttctgccttgtcatgctgatgggccagcccggcctggtgcatcaaactccaaagcccatgtggactgggccgacctgtattgatagctctacttataattatatgtcattatgttggtagtgttgattgtctggaagttaagtgaattatctgtctaatgtgaatagtgcttacatgttgaggtacttgtactgtcttatttgatttgtgggatattgttgtggttgtggaattattgcatcctcggtgatgttgacttagtttaattacatgaatgcacttttgtgtggctgactgaggcatgatgtggtactaagtcctttgtaaattaaggaaatatagcgtgcatggtcttggtaaattaaagttgaatgtcagagaacgtgttagtagttagtgaacttattacgttagaaatgaaatgataatgttaatcgaattaagaaagttaattgagaattaagtcgtagtaaggagtgtaaactttgatgctgaactgttatgtaagattgtcatgattgattttgttgattgtcatattgtgtttacatgaattaattttagtgagtcgataatacttatcattatgtgtggtatataaatactactctactatatcctttttggatgtaggtgtgatgcaggttgacttggagtttcttagttgggatggcgatgtactcttcttaacttttgcattgtattcaagcggaaggtgtgtctcttattatttattctcatttgtaaaagctcttgtacatgtctagccgaggtcttggatattgttgtaagatgttgagtgcaaacgcttgtaaataattttggtttataattaagttgatatacatatttaaactgcataactacataaaacatttacctaattttttattaaataaatggttggatggtaacagttctcctatctagttagaataaagcaggtgcacgacaatccagtgttttggattgtgacaatcctttcattctatttttaaaaaataattatttgatatgtgaagcctactgacacgactaatacgagttcactgcgcatgtctataaggattaaatgttgtagaatatccattaaacactttttggccctctttcttaaaaaataattgttttcatgaaggttaattccaaatttcacataaaatttcacttatgaaatatgaaagttcatgttgatgactatttttcaattataggactaattgaaaagtggctagttgtaaattttacttgtaatactgtaataaagagttttttcattcccagttttcaagttagaaatgttagaagaatctcaatcatctactgcaccttttggtggaggtgtagccaccttgatgcagggattcaaataatttttaccccttgctaaaaagttttacttattatacatgtattttatttgataaaaataataaaatgcatggtaaatttaacagttgaattccttcgaattgagatagaaccttgatctagcttatttatatatgaattgaatttcttaaataaaaattttgacttcgtcattaaggaaggtttagtcctcgcatttagttagtctcccaacacagatgaatcctataattaaacatagatgttgacgtttagccatagagagcaagcaagttagggtcaaagcatttgtggttaaatatcattctaataataggataattcccaattaataactaatggaccacatcaaaatacaacgcccagtcattcgtctatctctaacaaaaaaataactttttagaaaacatagcttataatggttacaaaaccaataaatggcggtatcagtacccatgaccagaaaacaactaagcccataaccatctggtagtcaccaaccagtcaaaaatcaactaccttctagtactcttctccctctaataaaaaactcacttcattcttctttataaaattccaaaaatacatttatttctctctgctaattttgttgatcagttttttctgtctaacgtttagaatcacacactctaagcaatcctccttaatatcgtgcataattattaatg

**Supplementary Fig. S5.** The 5 'non-coding sequence of genes related to anthocyanin synthesis of pepper. Motifs conformed to the known syntaxes are colored (suspected cis-form MREs in yellow block, suspected anti-form MREs in green block, suspected cis-form BREs in red font and suspected anti-form BREs in blue font).


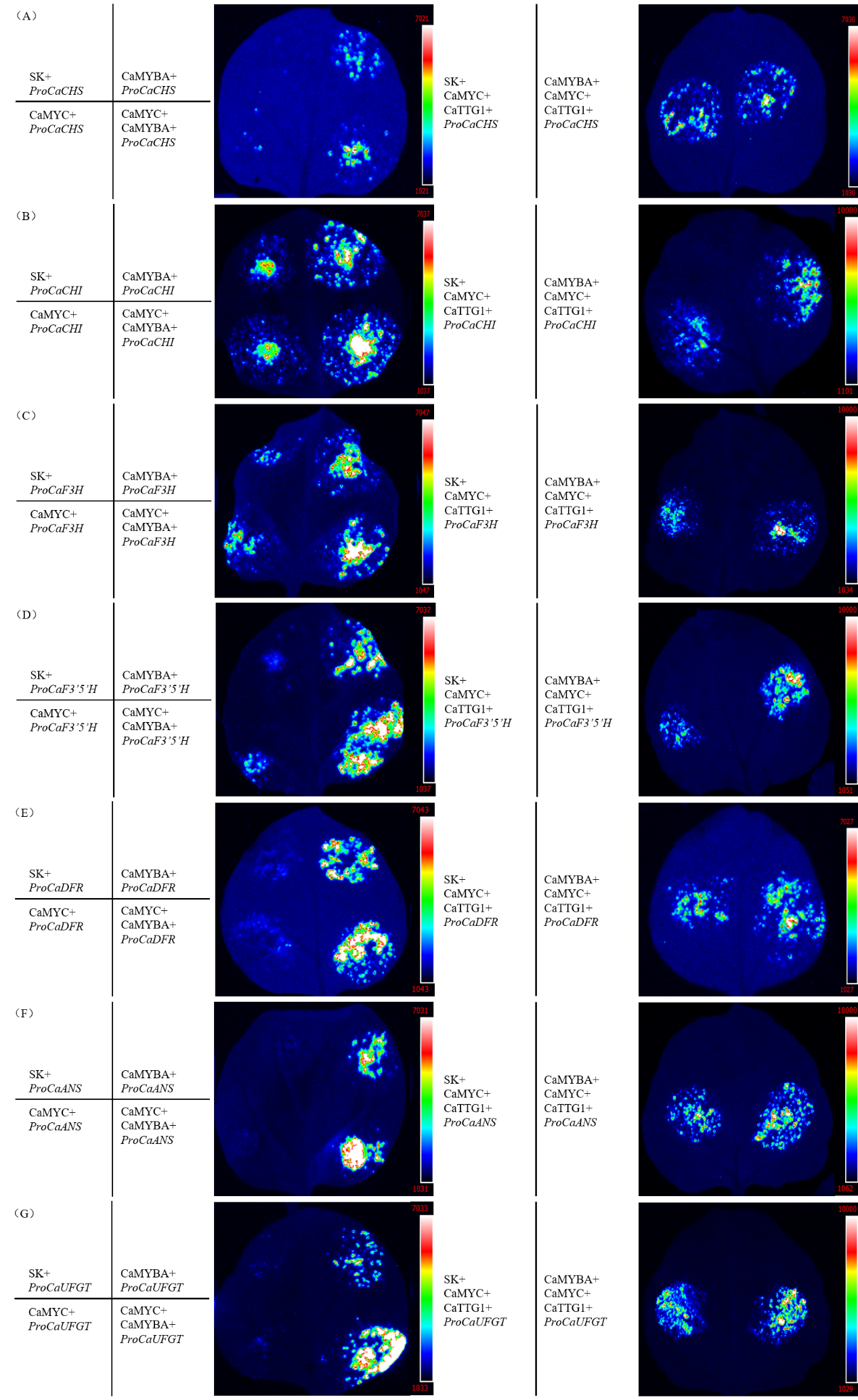


**Supplementary Fig. S6.** Double luciferase assay showed that CaMYBA alone or in combination with CaMYC and CaTTG1 could promote the expression of *CaCHS*, *CaCHI*, *CaF3H*, *CaF3'5'H*, *CaDFR*, *CaANS* and *CaUFGT*.


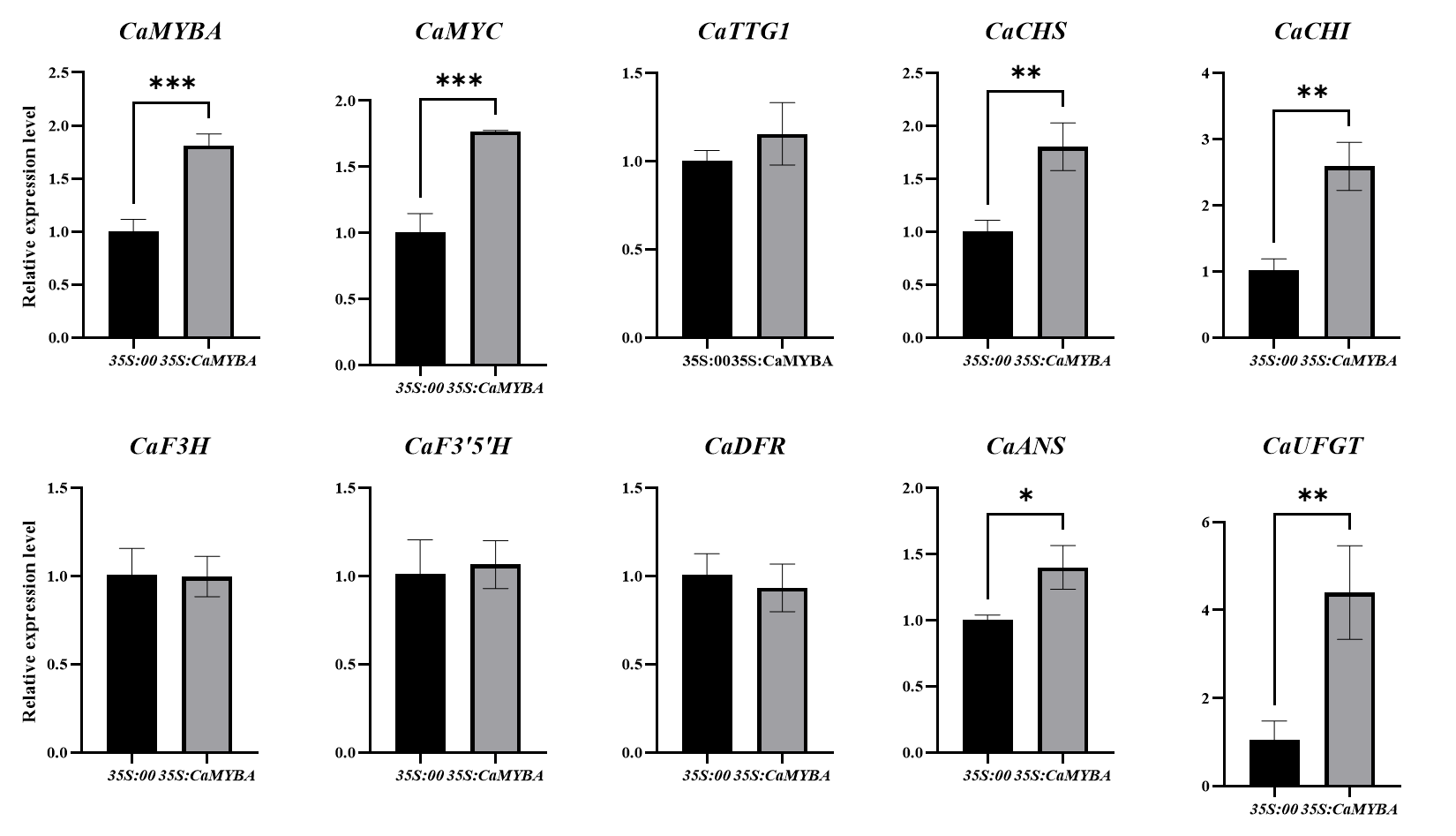


**Supplementary Fig. S7.** Detection of anthocyanin synthesis-related gene expression after transient overexpression of *CaMYBA* in pepper leaves. 35:00: negative control, 35S:CaMYBA: CaMYBA-overexpression. *CaUBI-3* was used as an internal control gene. Error bars represent the mean ± SD of three biological replicates.
